# Effect of H_2_O Adsorption on Negative Differential Conductance Behavior of Single Junction

**DOI:** 10.1038/s41598-017-04465-3

**Published:** 2017-06-23

**Authors:** Zong-Liang Li, Xiao-Hua Yi, Ran Liu, Jun-Jie Bi, Huan-Yan Fu, Guang-Ping Zhang, Yu-Zhi Song, Chuan-Kui Wang

**Affiliations:** School of Physics and Electronics, Shandong Normal University, Jinan, 250014 China

## Abstract

Large negative differential conductance (NDC) at lower bias regime is a very desirable functional property for single molecular device. Due to the non-conjugated segment separating two conjugated branches, the single thiolated arylethynylene molecule with 9,10-dihydroanthracene core (denoted as TADHA) presents excellent NDC behavior in lower bias regime. Based on the *ab initio* calculation and non-equilibrium Green’s function formalism, the NDC behavior of TADHA molecular device and the H_2_O-molecule-adsorption effects are studied systematically. The numerical results show that the NDC behavior of TADHA molecular junction originates from the Stark effect of the applied bias which splits the degeneration of the highest occupied molecular orbital (HOMO) and HOMO-1. The H_2_O molecule adsorbed on the terminal sulphur atom strongly suppresses the conductance of TADHA molecular device and destroys the NDC behavior in the lower bias regime. Single or separated H_2_O molecules adsorbed on the backbone of TADHA molecule can depress the energy levels of molecular orbitals, but have little effects on the NDC behavior of the TADHA molecular junction. Aggregate of several H_2_O molecules adsorbed on one branch of TADHA molecule can dramatically enhance the conductance and NDC behavior of the molecular junction, and result in rectifier behavior.

## Introduction

Utilizing single molecule as functional device in electronic circuit is an ultimate goal of molecular electronics, which has motivated scientists to devote themselves to the investigations of molecular devices for tens of years^1–10^. Due to the rapid development of single molecular technologies^11–14^, great progresses have been achieved for single-molecule-device fabrications in recent years^15–18^. At the meantime, different strategies are designed to control and improve the functional properties of single molecular device^19–22^. In order to gain insights into the controlling mechanism of single molecular functional characteristics^23–25^, the effects of external ambient^26–28^, electrode distance^29, 30^, molecule-electrode interface^31–35^, molecular anchor^36–43^, side group^44–47^, doping^48, 49^ and external field^50–55^ have been studied intensively. In experimental studies, molecular devices are often fabricated in solution and measured in vacuum or in gas circumstance, thus the effect of the surrounding molecule on the functional properties of molecular device should also be discussed^56–59^. Generally, the surrounding molecules play negative effects on the molecular junction, for example, aqueous solution or the water vapor can suppress the electronic transport of molecular junction dramatically^28, 56–58^. However, sometimes the surrounding molecules can also have positive effects, our recent research reveals that the small ambient molecules can make molecular junction more stable due to their suppressing the thermal vibrations of the molecular junction^14^. Therefore, studying the influence of surrounding molecules on molecular device is very helpful to improve the functional characteristics of the molecular device by properly applying surrounding molecules.

Due to the non-conjugated segment separating two conjugated arms, the single thiolated arylethynylene molecule with 9,10-dihydroanthracene core (denoted as TADHA) shows pronounced negative differential conductance (NDC) behavior, which has recent been detected by Perrin *et al*.^60^. Although the NDC behavior of single molecular device is often presented in theoretical studies, it is very scarce in experimental findings, and the NDC often shows very small for most cases. Thus, it is significant that Perrin *et al*. fabricated single TADHA molecular junction with large NDC behavior, especially the NDC behavior was occurred at lower bias regime because high bias voltage may deform the configuration of molecular junction^14^. Considering that the molecular junction was fabricated in solution, we are very interested in that if the junction is not absolutely dried, *i.e*., if one or several H_2_O molecules are left and adsorbed on the functional molecule, whether the NDC behavior can be destroyed or be enhanced? In order to answer this question, according to the density functional theory (DFT), we simulated the adsorptions of H_2_O molecules on different positions of the TADHA molecule. Then by applying non-equilibrium Green’s function (NEGF) formalism, the electronic transport properties of TADHA molecular junction were studied with the influences of H_2_O adsorbates. Our study shows that, the H_2_O molecules adsorbed on the terminal sulphur atoms not only destroy the NDC behavior of TADHA molecular device, but also depress the current of the molecular junction distinctly at lower bias regime. The NDC behavior of TADHA molecular device seems to show some water-immunity character for single or separated H_2_O molecules adsorbed on the backbone of TADHA molecule, but it is very sensitive to the aggregate of several H_2_O molecules. Our findings are valuable to the fabrication of TADHA molecular junction in solvent, *i.e*., one can determine whether there are H_2_O molecules adsorbed on the terminal S atoms or aggregate of H_2_O molecules adsorbed on the backbone of functional molecule by the electronic transport properties of molecular junction in experiment.

## Results

### One H_2_O molecule adsorption

**Figure 1:**
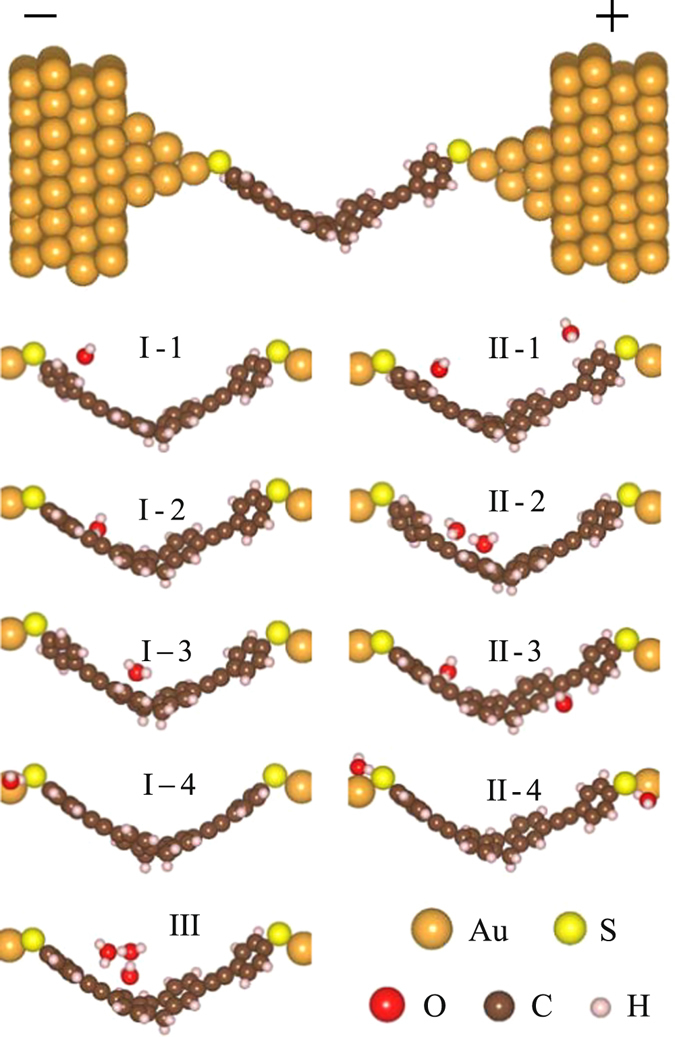
Schematic structures of TADHA molecular junctions with H_2_O molecule being adsorbed on different sites.

In order to simulate the TADHA molecular junction and study the H_2_O effect, we constructed Au-Molecule-Au systems by sandwiching TADHA molecule between two gold electrodes with one or several H_2_O molecules adsorbed on different sites of TADHA molecule as shown in Fig. 1. As for one-H_2_O-adsorption samples, based on the geometric optimizations, we found that there are four typical sites for H_2_O molecule to be adsorbed on, *i.e*., (1) the aryl ring site on one arm of TADHA molecule near the electrode, denoted as Type I-1; (2) the ethynylene site on one arm of TADHA molecule, denoted as Type I-2; (3) 9,10-dihydroanthracene core site, denoted as Type I-3; (4) the terminal sulphur atom site, denoted as Type I-4 (See Fig. 1). The calculations of ground-state-energy show that, the affinities for the H_2_O molecule adsorbed as Type I-1, 2, 3 and 4 are about 0.09, 0.2, 0.15 and 0.78 eV, respectively, which suggests that the H_2_O molecule is more likely to be adsorbed on the terminal S atoms, and then is the triple bond of TADHA molecule. The affinities also show that the adsorptions are very weak compared to the covalent bond.

**Figure 2:**
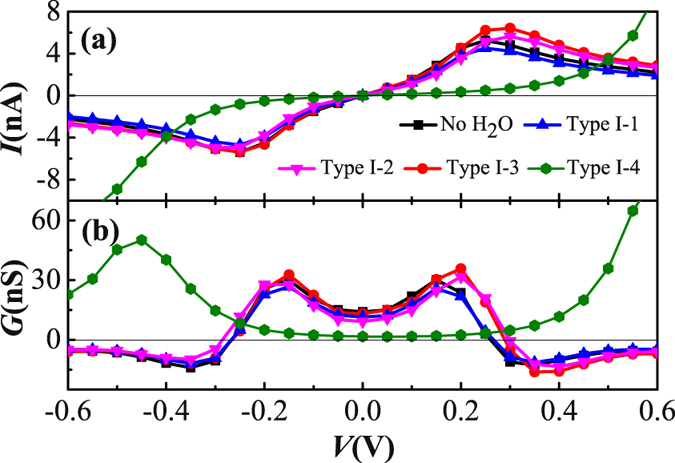
Electronic transport properties of TADHA molecular junction without or with one H_2_O molecule adsorbate, (**a**) Current and (**b**) Differential conductance as functions of applied bias voltage.

Figure 2 shows the current and the differential conductance as functions of applied bias for the TADHA molecular junctions without H_2_O adsorbate or with one H_2_O molecule being adsorbed on different sites of TADHA molecule. The figure shows that for the molecular junction without H_2_O molecule adsorbate, the molecular system shows symmetric electronic transport characters with respect to the positive and negative bias. Since the current shows peak values at about ±0.25 V, the NDC behavior appears in the differential conductance curve when the absolute value of the applied bias is larger than 0.25 V. The peak values of the NDC are presented at about ±0.35 V, which is a lower-bias NDC behavior as the experiment detected^60^. Since the molecular junction may be destroyed and the interface configuration may be deformed by high bias voltage and thermal vibration at room temperature^14^, one can expect that the low-bias NDC behavior is a very desirable function of molecular device in the future. When one H_2_O molecule is adsorbed on the TADHA molecule, the current and the differential conductance in the negative bias regime for the Type I-1, 2 and 3 molecular junctions show a little difference compared with those of the molecular junctions without the H_2_O adsorbate. However, in the positive bias regime for the voltage larger than 0.2 V, the TADHA molecular system seems more conductive with Type I-3 configuration (see Fig. 2). At the meantime, the peak values of the current and the differential conductance are shifted slightly to higher bias regime as well as the peak values of the NDC. It is noticeable that the NDC behavior of the molecular junction in the low bias regime is destroyed when the H_2_O is adsorbed on the terminal S site (Type I-4). At the same time, the low-bias current is depressed by about one order in the magnitude, which is similar to the molecular junctions probed by Long *et al*.^28^.

**Figure 3:**
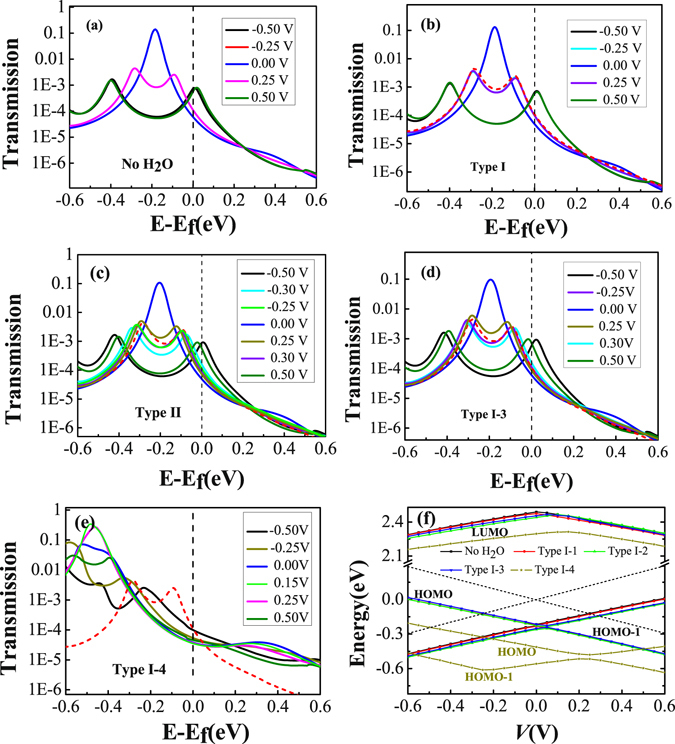
Transmission spectra and evolutions of the frontier molecular orbitals of TADHA molecular junctions. (**a**) Transmission spectra of TADHA molecular junction without H_2_O adsorbate; (**b**), (**c**), (**d**) and (**e**) Transmission spectra of TADHA molecular junction with one H_2_O adsorbate as Type I-1, 2, 3 and 4 molecular systems that are shown in Fig. 1. The red dashed lines in (**b**), (**c**) (**d**) and (**e**) are the transmission spectra of TADHA molecular junction without H_2_O adsorbate at 0.25 V bias voltage, which are used as reference curves in these four figures. (**f**) The energy of LUMO, HOMO and HOMO-1 as functions of bias voltage with one or without H_2_O adsorbate, the dashed line in (**f**) denotes bias windows.

In order to understand the NDC behavior of the TADHA molecular junction and the H_2_O-adsorbate effects, we presented the transmission spectra for the bias voltages of 0.0 V, ±0.25 V, ±0.5 V and the voltage of peak-current in Fig. 3. Due to the contributions of the highest occupied molecular orbital (HOMO) and HOMO-1 to the electronic transmission and the degenerate of these two molecular orbitals, a high transmission peak is presented at about −0.2 eV for the transmission curve with zero bias as Fig. 3(a) shows. When the bias is applied, the degenerate of the two molecular orbitals is destroyed by the Stark effect of the bias, which further splits the high transmission peak into two lower transmission peaks. Because of the split of the transmission peak, the transmission probability at Fermi energy level slightly increases with the increase of the bias in the lower bias regime. However, due to the rapid decrease of the height of the split transmission peaks with the increase of the bias, the area under the transmission curve in the bias window increases at first and reaches a peak value at about 0.25 V, and then decreases with the increase of the bias, which depends on the rivalry of the width of the bias window and the mean height of transmission spectra in bias window. Thus a current peak appears at about 0.25 V as well as the NDC behavior for the TADHA molecular junction without H_2_O adsorbate.

As for one H_2_O molecule adsorbed on the TADHA molecular junction, due to the strong electronegativity of the O atom, the TADHA molecular orbitals are modulated differently with the H_2_O molecule adsorbed on different positions, which further induces different changes of the transmission spectra as well as the conductance of the molecular system. In detail, for Type I-1 adsorption, since the H_2_O molecule is very close to the electrode and the affinity is very weak, the applied bias dominates the split of the energy levels and suppresses the effect of the H_2_O molecule by the electrodes, hence the transmission spectra show little difference from those of the TADHA molecular junction without H_2_O adsorbate (Fig. 3(b)). However, for Type I-2 and Type I-3 adsorption, the strong electronegativity of the O atom depresses the energy of the molecular orbital which close to it (Fig. 3(f)). Taking Type I-3 system as an example, since the H_2_O molecule is closer to the left branch of TADHA molecule, the energy of the orbital which locates on the left arm of TADHA molecule is lowered down, while the energy of the orbital which locates on the right branch of TADHA molecule is almost unaltered by the H_2_O molecule. As for positive bias, the HOMO is lowered, whereas the HOMO-1 is little influenced. Thus the transmission peaks are less separated with positive bias compared with that of the molecular junction without the H_2_O adsorbate. Consequently, the heights of the transmission peaks decrease a little more slowly in the split process with the increase of the positive bias than with negative bias, which can be easily seen by comparing the transmission curve of Type I-3 molecular system with that of the molecular system without H_2_O adsorbate at 0.25 V (Fig. 3(d)). Therefore, due to the effect of the H_2_O molecule on the HOMO and further on the transmission spectra, the TADHA molecular system with Type I-3 configuration shows more conductive when the bias is larger than 0.25 V, and the NDC behavior begins from a little higher bias voltage compared with that of the molecular system without H_2_O. On the contrary, for the negative bias, when the transmission peak is split, the H_2_O adsorbate is closer to HOMO-1, which results in a little red-shift of transmission peak corresponding to HOMO-1, while the transmission peak which corresponds to HOMO is almost unaltered. Since the HOMO-1 is shifted to the lower energy area and is apart from the bias window, one can easily understand why the current is changed very little by the H_2_O molecule in the negative bias regime. Different from the transmissions of Type I-1, 2 and 3 molecular systems, the transmission peaks are shifted to much lower energy regime by the strong influence of H_2_O adsorbate for Type I-4 system at lower bias. As Fig. 3(e) shows, the transmission peak related to the HOMO appears at about −0.4 eV, which is about 0.2 eV lower than those of other type molecular junctions. Thus, with lower bias, the transmissions of Type I-4 system in the bias windows are evidently smaller than the other cases, which results in the poor current of Type I-4 molecular junction in the lower bias regime. With the increase of the negative bias, the transmission peak corresponding to the HOMO is blue-shifted and gradually enters bias window (Fig. 3(f)). However, with the increase of the positive bias, the transmission peak corresponding to the HOMO is shifted very little. Therefore, the current begins increasing from relatively lower bias in the negative bias regime compared with positive bias.

**Figure 4:**
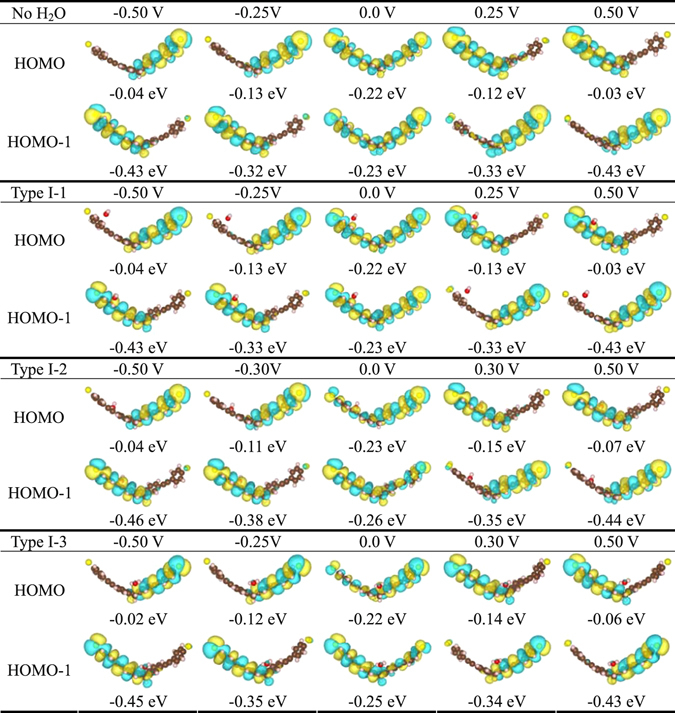
Spatial distributions of molecular orbitals for TADHA molecular junctions with one or without H_2_O adsorbate at 0.0 V, ±0.5 V and at the voltage of peak-current values, where the orbital energies relative to the Fermi level are also shown under each orbital.

In order to gain deep insight into the NDC behavior, we presented spatial distributions of the HOMOs and HOMOs-1 for TADHA molecular systems at 0.0 V, ±0.5 V and at the peak-current voltages in Fig. 4. The figure shows that, at 0.0 V, due to the approximate degeneration, the HOMO and HOMO-1 are both delocalized over the whole TADHA molecule not only for the molecular junction without H_2_O molecule, but also for the molecular junction with Type I-1, 2 and 3 configurations, which results in the high transmission peaks at about −0.2 eV for these molecular junctions at 0.0 V. However, there are still some differences for the effects of the H_2_O molecule on the orbital distributions at 0.0 V, such as for Type I-2 and 3 molecular systems. Attributing to the depressing of the H_2_O molecule, the gaps between the HOMO and HOMO-1 are slightly enlarged compared with the molecular system without the H_2_O molecule, and the spatial distributions of the orbitals are obviously asymmetric to the two branches of TADHA molecule. However, for Type I-1 molecular system, the orbitals are little influenced by the H_2_O molecule since the adsorption of H_2_O molecule is very weak. For the molecular junctions at peak-current voltage or at ±0.5 V, the HOMOs and the HOMOs-1 have been pulled apart by bias and each only locates on one branch of the TADHA molecule. Thus one can easily understand why the height of the transmission peaks decrease very quickly in the split process with the increase of the bias voltage. We should mention that the orbitals presented in the figure are the molecular projected self-consistent Hamiltonian (MPSH) eigenstates, which are often used to discuss the electronic transport properties of molecular junctions in literatures^7, 23^. The MPSH is the self-consistent Hamiltonian of the functional molecule including the H_2_O adsorbates with the influence of the electrode, which contains the electrode-molecule coupling effects but does not contain the Hamiltonian of the gold electrode, so the energies of the MPSH eigenstates are not perfectly consistent with the positions of the transmission peaks^7^.

### More H_2_O molecules or H_2_O aggregate adsorption

**Figure 5:**
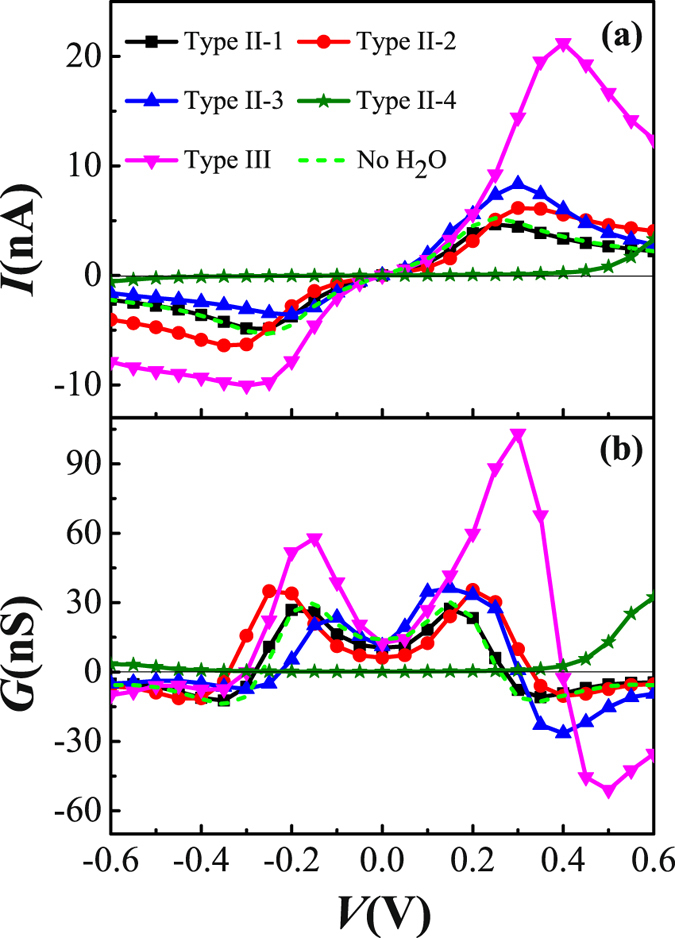
Electronic transport properties of TADHA molecular junction with two or three H_2_O molecule adsorbates. (**a**) Current and (**b**) Differential conductance as functions of applied bias voltage.

**Figure 6:**
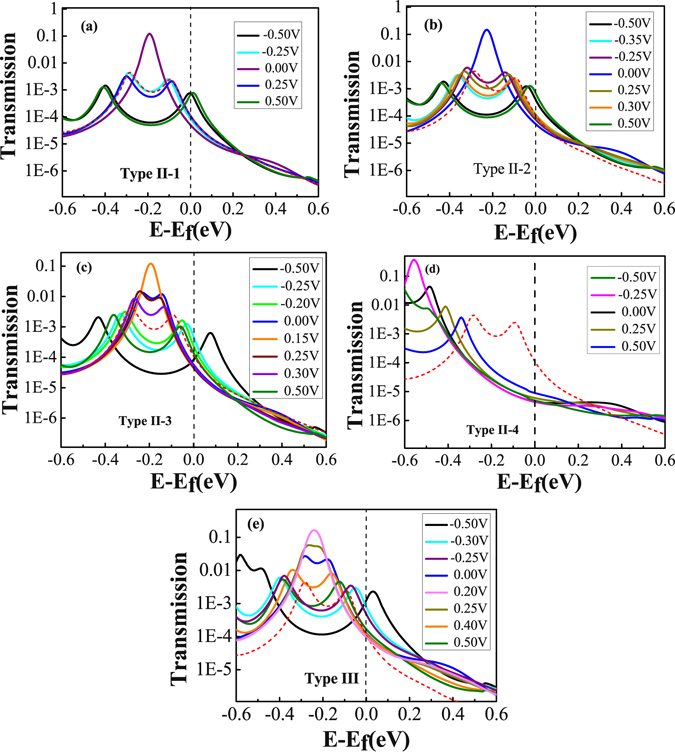
Transmission spectra of TADHA molecular junctions. (**a**), (**b**), (**c**) and (**d**) Transmission spectra for the molecular junction with two H_2_O adsorbates as Type II-1, 2, 3 and 4 molecular systems; (**e**) Transmission spectra for the molecular junction with three aggregated H_2_O molecules being adsorbed on as Type III molecular systems. The red dashed lines in (**a**), (**b**), (**c**), (**d**) and (**e**) are the transmission spectra of TADHA molecular junction without H_2_O adsorbate at 0.25 V bias voltage, which are used as reference curves in the figure.

In order to understand the effect of H_2_O molecules on the electronic transports more explicitly, we further set two or more H_2_O molecules adsorbed on the TADHA molecule, which are shown in Fig. 1, where Type II-1, 2 and 3 are the molecular junctions with two H_2_O molecules being adsorbed on TADHA molecule, Type III is the molecular junction with three H_2_O adsorbates. After geometric optimizations, we calculated the electronic transport properties for the molecular systems with Type II and Type III configurations. Figure 5 shows the current and the differential conductance of TADHA molecular junctions with two or three H_2_O adsorbates as functions of applied bias. From the figure one can see that, TADHA molecular junctions still show low-bias NDC behaviors with the influence of two or three H_2_O molecules adsorbed on the backbone of TADHA molecule. For the Type II-1 and Type II-2 molecular junctions, the electronic transport properties show similar characteristics to Type I molecular systems. In detail, due to the influences of the H_2_O molecules adsorbed near the electrodes being suppressed by bias voltage, the electronic transport properties of Type II-1 (see Figs 5 and 6(a)) are very similar to those of Type I-1 molecular system and the molecular system without H_2_O adsorbate. For the Type II-2 molecular system, since the H_2_O molecule adsorbed on the ethynylene site can depress the energy of the frontier molecular orbital which close to the H_2_O molecule and consequently induces red-shift of the transmission peaks, the peak value of the current is shifted to higher bias voltage compared to the ones without H_2_O adsorbate (see Figs 5 and 6(b)). Since the influences of the two H_2_O molecules are nearly symmetric to the TADHA molecular junctions, the current curves and the conductance curves are both approximately symmetric to the positive and negative bias for the junctions with Type II-1 and Type II-2 configuration. Attributing to the approximately symmetric effects of the two H_2_O molecules, at 0.0 V, the spatial distributions of HOMO and HOMO-1 for Type II-1 and Type II-2 molecular systems also present approximately symmetric character to the two branches of the TADHA molecule (Figure S1).

Our calculations show that, if we first set two H_2_O molecules on the two conjugated rings of the 9,10-dihydroanthracene core respectively and perform geometric optimization, the two H_2_O molecules will move and aggregate with each other, until at last formed Type II-3 configuration, which obviously due to the electrostatic attraction originated from the strong polarity of H_2_O molecule. From Fig. 5 one can see that, the current and the differential conductance curves of Type II-3 system are more asymmetric than those of Type I-3. The peak current value is further enhanced and the peak NDC value is more than doubled of that without H_2_O adsorbate for the junction in the positive bias regime, whereas for the negative bias, which is obviously depressed by the influence of the H_2_O adsorbates. Thus, for TADHA molecular junction with Type II-3 configuration, the adsorption of H_2_O molecules induces apparent rectifier behavior with the maximum rectification ratio of 2.74 at 0.35 V.

Figure 6(c) shows that, for Type II-3 molecular system, due to the depressing of the two H_2_O molecules to the molecular orbitals, the HOMO and HOMO-1 are not degenerated at zero bias, thus the transmission spectrum shows two peaks at about −0.15 eV and −0.24 eV corresponding to the HOMO and HOMO-1. With the increase of the positive bias, the HOMO is depressed and simultaneously the HOMO-1 is enhanced. When the bias is increased to about 0.15 V, the HOMO and HOMO-1 are re-degenerated, which results in the combination of the two transmission peaks into one higher transmission peak at 0.19 eV. Hence, the TADHA molecular junction with Type II-3 configuration is more conductive in the positive bias regime. Different from positive bias, the negative bias further splits and depresses the two transmission peaks which makes the molecular junction less conductive when the bias less than −0.20 V, so the TADHA molecular junction shows rectifier behavior. In fact, from the asymmetric evolution of the HOMO and HOMO-1, especially, the degeneration point of the HOMO and HOMO-1 obviously deviates from 0.0 V (Figure S2), one can also understand the rectifier behavior of Type II-3 molecular system, because the degeneration of the HOMO and HOMO-1 results in the delocalization of the HOMO and HOMO-1 (Figure S1), and consequently enhances the current of positive bias regime.

Similar to one-H_2_O-molecule adsorption on S atom site, for the Type II-4 molecular system that each terminal S atom adsorbs one H_2_O molecule, the NDC behavior of TADHA molecular junction has been destroyed absolutely in the lower bias regime. Due to the strong influence of the adsorbates, the conducting orbitals have been further depressed to lower than −0.5 eV (Fig. 6(d) and Figure S1), which results in much poorer conductance of Type II-4 molecular system compared with the molecular system without H_2_O adsorbate or with one H_2_O molecule adsorbed on the terminal S atom. The numerical results show that, for the bias lower than 0.25 V, the current of Type II-4 molecular system is about two orders lower than that of the system without the H_2_O adsorbate in the magnitudes, which is in good agreement with Long’s experiment^28^. Although with the increase of the positive bias, the transmission peak corresponding to the HOMO is shifted to higher energy regime, it is still out of the bias window when the bias is enhanced to 0.5 V (Figure S2). So the current is very weak for the Type II-4 molecular junction.

## Discussion

It is interesting that, for the H_2_O molecules being adsorbed on the backbone of TADHA molecule, the aggregate of H_2_O molecules seems to have stronger influence on the electronic transport properties of TADHA molecular junction than single or separated H_2_O molecules. Especially, with the influence of the aggregate of H_2_O molecules adsorbed on one branch of TADHA molecule, the values of the current and NDC peaks are both enhanced dramatically. Taking the aggregate of three H_2_O molecules as an example, after geometric optimization we obtained the configuration of Type III molecular junction (Fig. 1). From Fig. 5 one can see that the peak-current value is up to about 21 nA, which is more than four times than that for the system without H_2_O adsorbate. Similar to Type II-3 molecular system, the depression of the H_2_O aggregate destroys the degeneration of the HOMO and HOMO-1 at zero bias, and consequently splits the high transmission peak at about 0.2 eV (Fig. 6(e)). Since the transmission spectra near Fermi energy is changed very slightly, the current is slightly changed in the lower bias regime. However, when the HOMO and HOMO-1 are re-degenerated at the positive bias of about 0.2 V, the two transmission peaks which are related to the HOMO and HOMO-1 are combined into one high peak. Further enhancing the positive bias after the two orbitals re-degeneration, the combined transmission peak is re-split, and compared with former cases, the transmission peak corresponding to HOMO enters bias window with a higher height. For example, at 0.25 V and 0.4 V, the height of the transmission peak related to the HOMO is about 0.05 and 0.01, respectively. However, except for the Type II-3 with an aggregate of two H_2_O molecules, the heights of the transmission peaks corresponding to the HOMO are all no more than 0.004 for the other cases at 0.25 V, not to mention at 0.4 V. Thus, when the currents of other cases reach the peak values at about 0.25–0.30 V, the current of Type III system still increases rapidly until reaches a much higher current peak at 0.4 V. Then due to the rapid shrink of the transmission peak which is induced by the further split of the HOMO and the HOMO-1, the current decreases quickly and results in larger NDC behavior.

As to the accuracy of our work, we found that our calculations successfully reproduced the low-bias NDC behavior of TADHA molecular junction that was investigated experimental by Perrin *et al*.^60^. However, the absolute values of the current in the lower bias regime are about one order of magnitude larger than the experimental results. According to the studies of Quek *et al*.^61–63^, the HOMO-LUMO (LUMO: lowest unoccupied molecular orbital) gap is usually underestimated in standard density functional theory (DFT), which results in overestimate of conductance of single molecular junction. Thus an alternative DFT-based approach^61–65^ (e.g. DFT + Σ approach) is needed to give more accurate and physically meaningful understandings of electron-transport properties of TADHA molecular junction in the future. Since the HOMO is much closer to the Fermi level than the LUMO, the electron-transport properties are mainly governed by the HOMO. According to the DFT + Σ method in the literatures^61–63^ and the experimental results^60^, we can roughly estimate that, for the TADHA molecular junction without H_2_O adsorbate, the HOMO is overestimated by not more than 0.3 eV relative to the Fermi level, which is much smaller than those of weak coupling systems^61–63^.

In summary, the NDC behavior of TADHA molecular junctions and the H_2_O adsorption effect are investigated by applying the density-functional theory and non-equilibrium Green’s function method. In the lower bias regime, the TADHA molecular junction exhibits excellent NDC behavior, which is attributed to the splitting of the degenerated transmission channels at applied bias voltage. The H_2_O molecules may depress the energy of the transmission channel, and further influence the electronic transport properties of the molecular junction. The influences of the H_2_O molecules are closely related to the H_2_O molecule affinity. Although the single H_2_O molecule or the separated H_2_O molecules adsorbed on the backbone of TADHA molecule can lower the energies of the TADHA molecular orbitals and shift the positions of the transmission peaks, they have relatively smaller effects on the current and NDC behavior of the TADHA molecular junction. The aggregate of several H_2_O molecules adsorbed on one branch of the TADHA molecule can strongly enhance the current and the NDC behavior of the molecular junction. The H_2_O molecule adsorbed on the terminal S atom strongly depresses the conductive orbitals, which further dramatically suppresses the current of TADHA molecular junction in the lower bias regime and consequently destroys the low-bias NDC behavior of the molecular junction.

## Methods

The geometric structures of TADHA molecular systems without or with H_2_O molecules were optimized using the SIESTA package with a maximum force 0.02 eV/Å^66, 67^. The Troullier–Martin type norm-conserving pseudopotentials are applied to represent the core electrons^68^, the generalized gradient approximation (GGA) with Perdew–Burke–Ernzerhof (PBE) formulation is applied as the exchange-correlation functional^69^. For Au atoms, a single-*ζ* plus polarization basis set is used, and for other atoms, a double-*ζ* plus polarization basis set is employed.

The current through the molecular device with different bias voltage is obtained according to the Landauer–Buttiker formula^70^

1 $$I=\frac{2e}{h}\int T(E,V)\,[f(E-{\mu }_{L})-f(E-{\mu }_{R})]dE,$$

which was calculated with the TranSIESTA module of the SIESTA package. In Eq. (1), *T*(*E*, *V*) is the transmission probability, which depends on the incident energy *E* of the transmission electrons and the applied bias voltage *V*. ${\mu }_{L}$ and ${\mu }_{R}$ in the Fermi–Dirac distribution functions *f*(*E*) are the electrochemical potentials of the two electrodes. The transmission probability *T*(*E*, *V*) is calculated by NEGF method. The differential conductance is defined as $G=\partial I/\partial V$. In the electron transport calculations, a 300 Ry mesh cutoff for the real space grid was chosen. The convergence criterion for density matrix was set to 1.0 × 10^−4^. A 4 × 4 *k*-point grid was used for the Brillouin-zone (BZ) sampling in the transverse directions. A 300 K smearing was applied for the electronic Fermi-Dirac distribution.

## Electronic supplementary material

Supplementary Information

## References

[1] Xiang D, Wang XL, Jia CC, Lee T, Guo XF (2016). Molecular-scale electronics: from concept to function. Chem. Rev..

[2] Wang CK, Luo Y (2003). Current–voltage characteristics of single molecular junction: dimensionality of metal contacts. J. Chem. Phys..

[3] Jiang J, Kula M, Luo Y (2006). A Generalized quantum chemical approach for elastic and inelastic electron transports in molecular electronics devices. J. Chem. Phys..

[4] Zhang XJ, Chen KQ, Tang LM, Long MQ (2011). Electronic transport properties on V-shaped-notched zigzag graphene nanoribbons junctions. Phys. Lett. A.

[5] Liu R, Wang CK, Li ZL (2016). A method to study electronic transport properties of molecular junction: one-dimension transmission combined with three-dimension correction approximation (OTCTCA). Sci. Rep..

[6] Dou KP, Fu XX, De Sarkar A, Zhang RQ (2016). Dual response of graphene-based ultra-small molecular junctions to defect engineering. Nano Res..

[7] Zhang ZH (2013). A Dramatic Odd-Even Oscillating Behavior for the Current Rectification and Negative Differential Resistance in Carbon-Chain-Modified Donor-Acceptor Molecular Devices. Adv. Funct. Matr..

[8] Hu GC, Zhang Z, Li Y, Ren JF, Wang CK (2016). Length dependence of rectification in organic co-oligomer spin rectifiers. Chin. Phys. B.

[9] Zhang XJ, Chen KQ, Long MQ, He J, Gao YL (2015). Effect of length and negative differential resistance behavior in conjugated molecular wire tetrathiafulvalene devices. Mod. Phys. Lett. B.

[10] Li ZL (2011). Theoretical study on electronic transport properties of oligothiophene molecular devices. Chin. J. Chem. Phys..

[11] Cui XD (2001). Reproducible measurement of single-molecule conductivity. Science.

[12] Sun M, Zhang Z, Kim ZH, Zheng H, Xu H (2013). Plasmonic scissors for molecular design. Chem. Eur. J..

[13] Xiang D, Jeong H, Lee T, Mayer D (2013). Mechanically controllable break junctions for molecular electronics. Adv. Mater..

[14] Wang Q (2016). Single-atom switches and single-atom gaps using stretched metal nanowires. ACS Nano.

[15] Taniguchi M, Tsutsui M, Shoji K, Fujiwara H, Kawai T (2009). Single-molecule junctions with strong molecule-electrode coupling. J. Am. Chem. Soc..

[16] Taniguchi M (2011). Dependence of single-molecule conductance on molecule junction symmetry. J. Am. Chem. Soc..

[17] Xiang D, Zhang Y, Pyatkov F, Offenhäusser A, Mayer D (2011). Gap size dependent transition from direct tunneling to field emission in single molecule junctions. Chem. Commun..

[18] Xu BQ, Tao NJ (2003). Measurement of single-molecule resistance by repeated formation of molecular junctions. Science.

[19] Liu R (2014). Study on force sencitivity of electronic transport properties of 1,4-butanedithiol molecular device. Acta Phys. Sin..

[20] Li Y, Feng Y, Sun M (2015). Photoinduced charge transport in a BHJ solar cell controlled by an external electric field. Sci. Rep..

[21] Zhang YF, Yi XH, Zhang Z, Sun JX, Li ZL (2015). Theoretical studies on electronic transport properties of 2,5-dimercapto-pyridazin molecular junctions: influence of CO and H_2_O molecules. J. At. Mol. Sci..

[22] Xiang D, Lee T, Kim Y, Mei TT, Wang QL (2014). Origin of discrete current fluctuations in a single molecule junction. Nanoscale.

[23] Song Y, Xie Z, Ma Y, Li ZL, Wang CK (2014). Giant Rectification Ratios of Azulene-like Dipole Molecular Junctions Induced by Chemical Doping in Armchair-Edged Graphene Nanoribbon Electrodes. J. Phys. Chem. C.

[24] Zhao WK (2015). Rectification inversion in oxygen substituted graphyne–graphene-based heterojunctions. Phys. Chem. Chem. Phys..

[25] Zou D, Zhao W, Fang C, Cui B, Liu D (2016). The electronic transport properties of zigzag silicene nanoribbon slices with edge hydrogenation and oxidation. Phys. Chem. Chem. Phys..

[26] Li ZL, Li HZ, Ma Y, Zhang GP, Wang CK (2010). Hydration effect on the electronic transport properties of oligomeric phenylene ethynylene molecular junctions. Chin. Phys. B.

[27] Lin XN, Zhang GP, Ren JF, Yuan XB, Hu GC (2014). Electronic transport properties of oligophenyleneethynylene molecular junctions in alkaline and acid solutions. Acta Phys. Sin..

[28] Long DP (2006). Effects of hydration on molecular junction transport. Nature Mater..

[29] Yi XH (2016). Low-Bias negative differential conductance controlled by electrode separation. Chin. Phys. B.

[30] Zhang GP, Hu GC, Song Y, Xie Z, Wang CK (2013). Stretch or contraction induced inversion of rectification in diblock molecular junctions. J. Chem. Phys..

[31] Zhao WK, Ji GM, Liu DS (2014). Contact position and width effect of graphene electrode on the electronic transport properties of Dehydrobenzoannulenne molecule. Phys. Lett. A.

[32] Li ZL, Zou B, Wang CK, Luo Y (2006). Electronic transport properties of molecular bipyridine junctions: effects of isomer and contact structures. Phys. Rev. B.

[33] Li Y (2016). Spin Polarization at Organic-Ferromagnetic Interface: Effect of Contact Configuration. Chin. J. Chem. Phys..

[34] Jiang ZL, Wang H, Shen ZY, Sanvito S, Hou SM (2016). Effects of the molecule-electrode interface on the low-bias conductance of Cu-H2-Cu single-molecule junctions. J. Chem. Phys..

[35] Hu GC (2015). Effect of interfacial coupling on the rectification in organic spin rectifiers. Chin. Phys. B.

[36] Li MJ, Xu H, Chen KQ, Long MQ (2012). Electronic transport properties in benzene-based heterostructure: Effects of anchoring groups. Phys. Lett. A.

[37] Hong W (2012). Single molecular conductance of tolanes: experimental and theoretical study on the junction evolution dependent on the anchoring group. J. Am. Chem. Soc..

[38] Hong W (2012). Trimethylsilyl-terminated oligo(phenylene ethynylene)s: an approach to single-molecule junctions with covalent Au−C σ-Bonds. J. Am. Chem. Soc..

[39] Bao DL (2014). Theoretical study on mechanical and electron-transport properties of conjugated molecular junctions with carboxylic or methyl sulfide links. Phys. Lett. A.

[40] Li XL (2006). Conductance of single alkanedithiols: Conduction mechanism and effect of molecule-electrode contacts. J. Am. Chem. Soc..

[41] Chen F, Li X, Hihath J, Huang Z, Tao N (2006). Effect of anchoring groups on single-molecule conductance: comparative study of thiol-, amine-, and carboxylic-acid-terminated molecules. J. Am. Chem. Soc..

[42] Park YS (2007). Contact chemistry and single-molecule conductance: A comparison of phosphines, methyl sulfides, and amines. J. Am. Chem. Soc..

[43] Yokota K, Taniguchi M, Tsutsui M, Kawai T (2010). Molecule-electrode bonding design for high single-molecule conductance. J. Am. Chem. Soc..

[44] Fu XX, Zhang LX, Li ZL, Wang CK (2013). Switching properties of bi-OPE-monothiol molecular junctions: Substituent effects and improvement of open-close ratio. Chin. Phys. B.

[45] Xiang D (2011). Molecular Junctions Bridged by Metal Ion Complexes. Chem. Eur. J..

[46] Fu XX, Zhang RQ, Zhang GP, Li ZL (2014). Rectifying properties of oligo(phenylene ethynylene) heterometallic molecular junctions: molecular length and side group effects. Sci. Ref..

[47] Di Ventra M, Kim SG, Pantelides ST, Lang ND (2001). Temperature effects on the transport properties of molecules. Phys. Rev. Lett..

[48] Zou DQ, Song Y, Xie Z, Li ZL, Wang CK (2015). Large rectification ratio induced by nitrogen (boron) doping in graphene nanoribbon electrodes for OPE junctions. Phys. Lett. A.

[49] Yang Z, Lang ND, Di Ventra M (2003). Effects of geometry and doping on the operation of molecular transistors. Appl. Phys. Lett..

[50] Xu BQ, Xiao XY, Yang XM, Zang L, Tao NJ (2005). Large gate modulation in the current of a room temperature single molecule transistor. J. Am. Chem. Soc..

[51] Li ZL, Fu XX, Zhang GP, Wang CK (2013). Effect of gate electric field on single organic molecular devices. Chin. J. Chem. Phys..

[52] Su W, Jiang J, Lu W, Luo Y (2006). First-Principles Study of Electrochemical Gate-Controlled Conductance in Molecular Junctions. Nano Lett..

[53] Xu BQ, Li XL, Xiao XY, Sakaguchi H, Tao NJ (2005). Electromechanical and conductance switching properties of single oligothiophene molecules. Nano Lett..

[54] Li ZL, Zhang GP, Wang CK (2011). First-principles study on formation and electron transport properties of single oligothiophene molecular junctions. J. Phys. Chem. C.

[55] Xiang D (2013). Three-terminal single-molecule junctions formed by mechanically controllable break junctions with side gating. Nano Lett..

[56] Li XL (2007). Thermally activated electron transport in single redox molecules. J. Am. Chem. Soc..

[57] Na JS, Ayres J, Chandra KL, Gorman CB, Parsons GN (2007). Real-time conductivity analysis through single-molecule electrical junctions. Nanotechnology.

[58] Cao H, Jiang J, Ma J, Luo Y (2008). Temperature-dependent statistical behavior of single molecular conductance in aqueous solution. J. Am. Chem. Soc..

[59] Cao H, Ma J, Luo Y (2010). Field effects on the statistical behavior of the molecular conductance in a single molecular junction in aqueous solution. Nano Res..

[60] Perrin ML (2014). Large negative differential conductance in single-molecule break junctions. Nature Nanotech..

[61] Quek SY, Choi HJ, Louie SG, Neaton JB (2009). Length dependence of conductance in aromatic single-molecule junctions. Nano Lett..

[62] Quek SY (2007). Amine-gold linked single-molecule circuits: experiment and theory. Nano Lett..

[63] Quek SY, Khoo KH (2014). Predictive DFT-based approaches to charge and spin transport in single-molecule junctions and two-dimensional materials: successes and challenges. Acc. Chem. Res..

[64] Koentopp M, Burke K, Evers F (2006). Zero-bias molecular electronics: Exchange-correlation corrections to Landauer’s formula. Phys. ReV. B.

[65] Darancet P, Ferretti A, Mayou D, Olevano V (2007). *Ab initio* GW electron-electron interaction effects in quantum transport. Phys. ReV. B.

[66] Brandbyge M, Mozos JL, Ordejόn P, Taylor J, Stokbro K (2002). Density-functional method for nonequilibrium electron transport. Phys. Rev. B.

[67] Soler JM (2002). The SIESTA method for ab initio order-N materials simulation. J. Phys.: Condens. Matter.

[68] Troullier N, Martins JL (1990). A straightforward method for generating soft transferable pseudopotentials. Solid State Commun..

[69] Perdew JP, Burke K, Ernzerhof M (1996). Generalized Gradient Approximation Made Simple. Phys. Rev. Lett..

[70] Buttiker M, Imry Y, Landauer R, Pinhas S (1985). Generalized many-channel conductance formula with application to small rings. Phys. Rev. B.

