# Supplementary material for: Effect of H2O Adsorption on Negative Differential Conductance Behavior of Single Junction
Source: Sci Rep. 2017 Jun 23;7:4195. doi: 10.1038/s41598-017-04465-3 (PMC5482906; doi:10.1038/s41598-017-04465-3)
Supplement: Supplementary file 1 — Supplementary Information [file 41598_2017_4465_MOESM1_ESM.doc]

**Effect of H_2_O Adsorption on Negative Differential Conductance Behavior of Single Molecular Junction**

Zong-Liang Li^*^, Xiao-Hua Yi, Ran Liu, Jun-Jie Bi, Huan-Yan Fu, Guang-Ping Zhang, Yu-Zhi Song, Chuan-Kui Wang

School of Physics and Electronics, Shandong Normal University, Jinan, 250014, China

^*^Correspondence and requests for materials should be addressed to Z. L. Li ([lizongliang@sdnu.edu.cn](mailto:lizongliang@sdnu.edu.cn))

**Supplementary Information**

1. Spatial distribution of TADHA molecular junctions.

| Type I-4 | -0.50 V | -0.25V | **0.0 V** | 0.25 V | 0.50 V |
| --- | --- | --- | --- | --- | --- |
| HOMO | 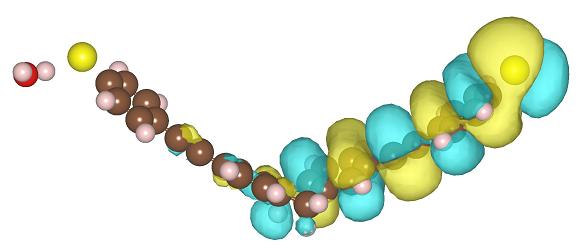 | 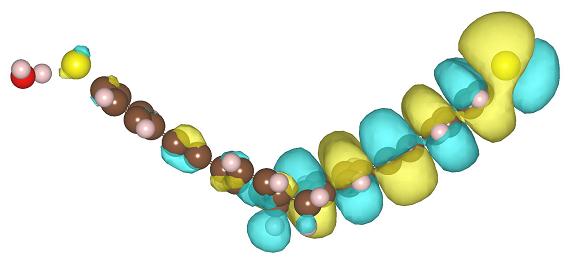 | **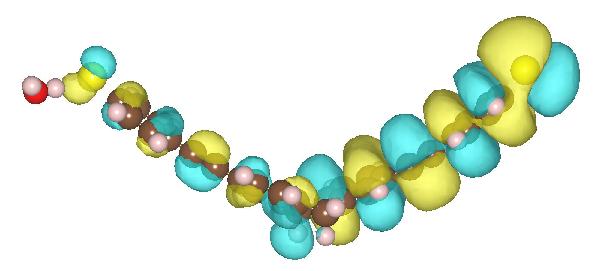** | 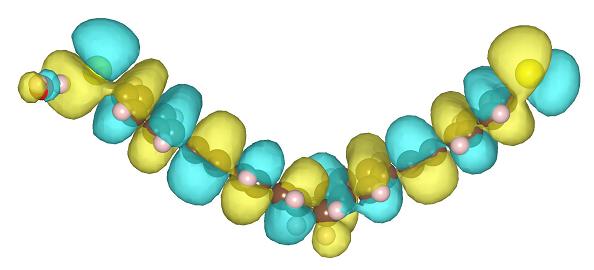 | 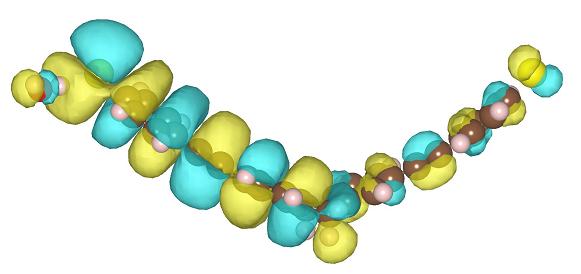 |
|  | -0.242eV | -0.326eV | **-0.413eV** | -0.48eV | -0.43eV |
| HOMO-1 | 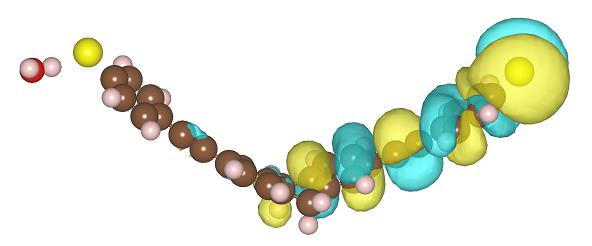 | 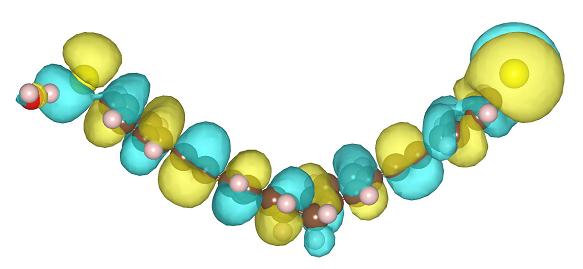 | **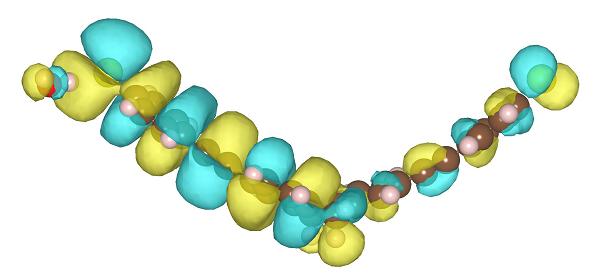** | 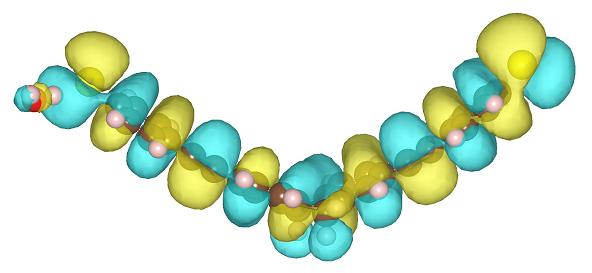 | 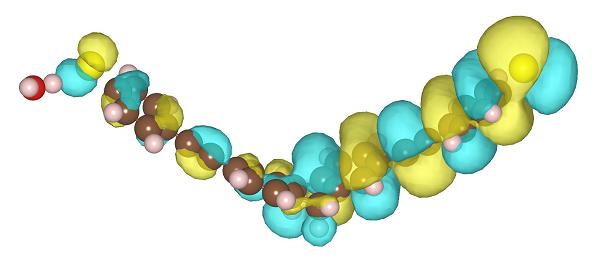 |
|  | -0.508eV | -0.612eV | **-0.559eV** | -0.517eV | -0.597eV |
| Type II-1 | -0.50 V | -0.25V | **0.0 V** | 0.25 V | 0.50 V |
| HOMO | 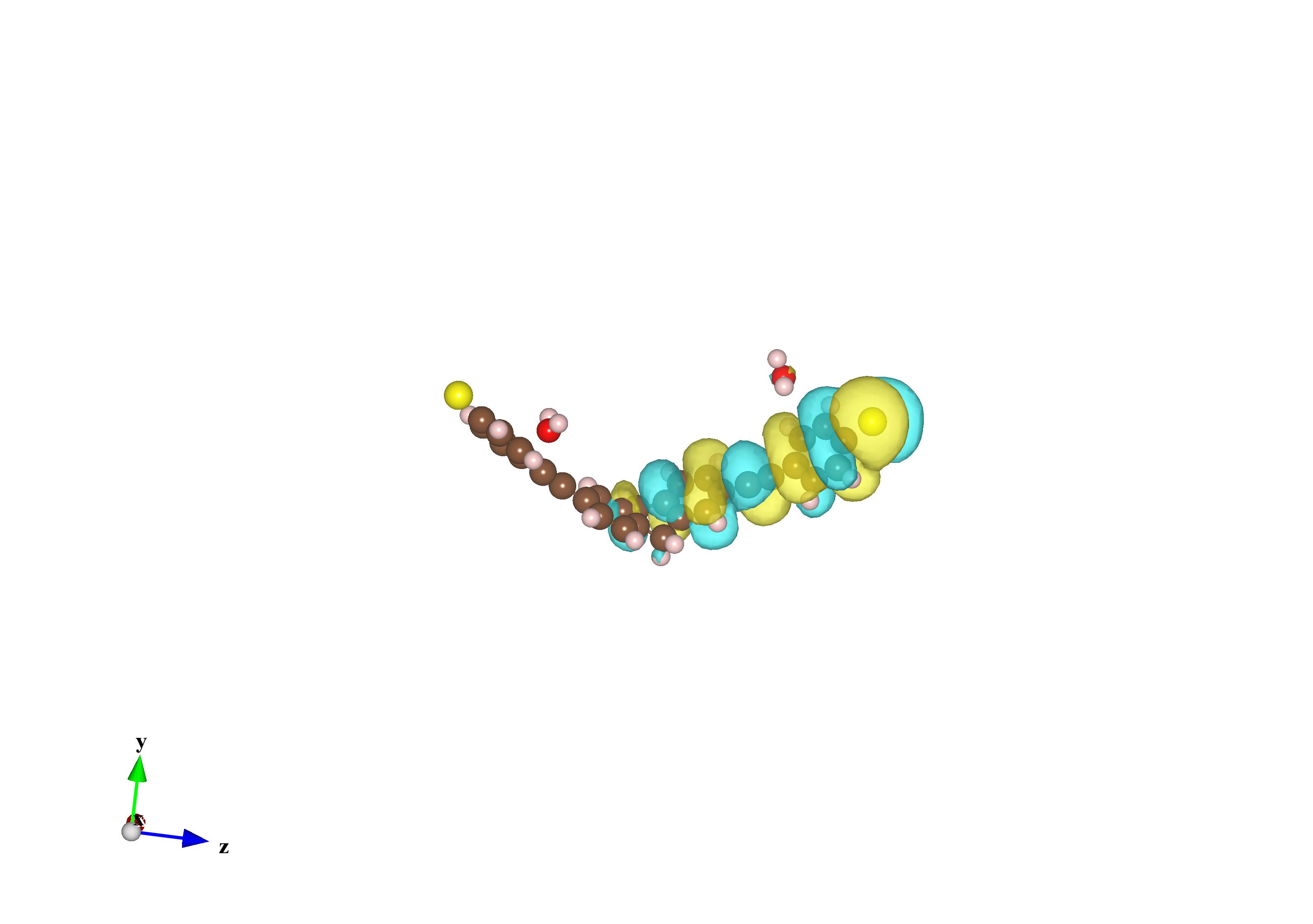 | 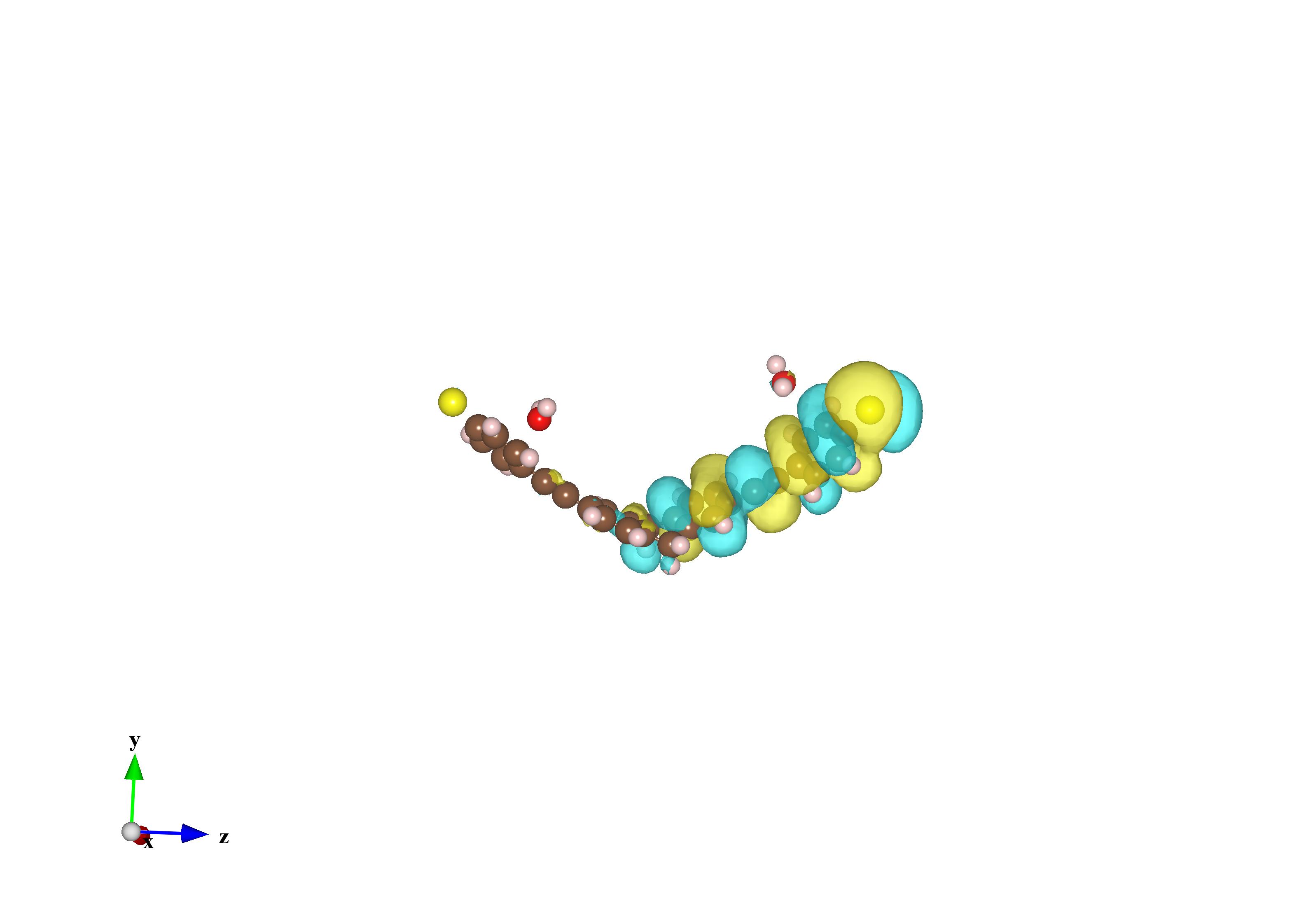 | **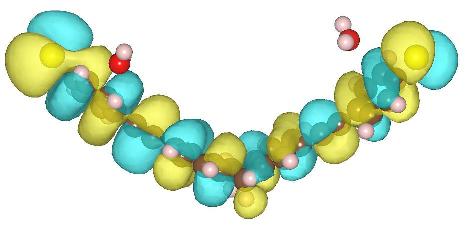** | 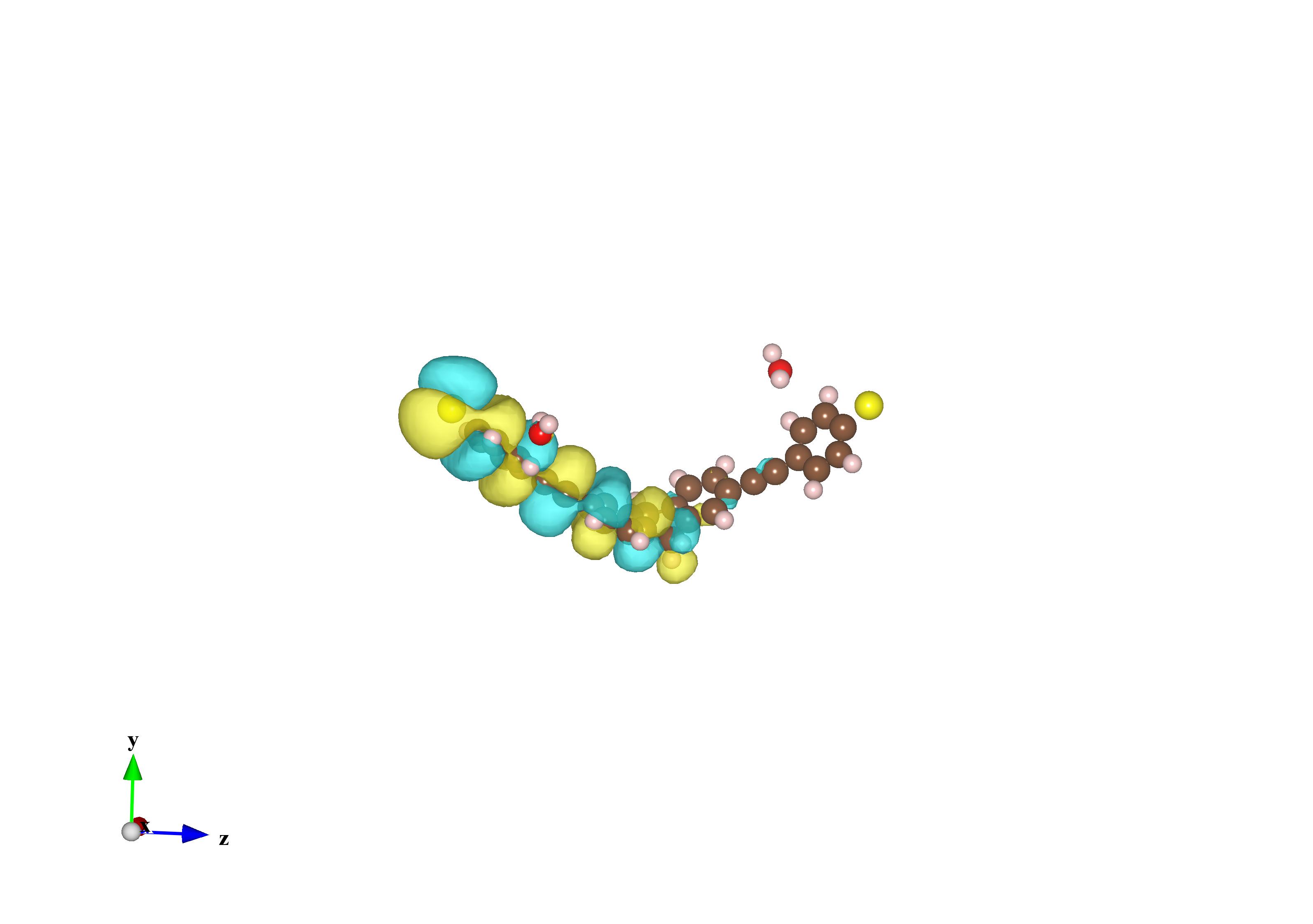 | 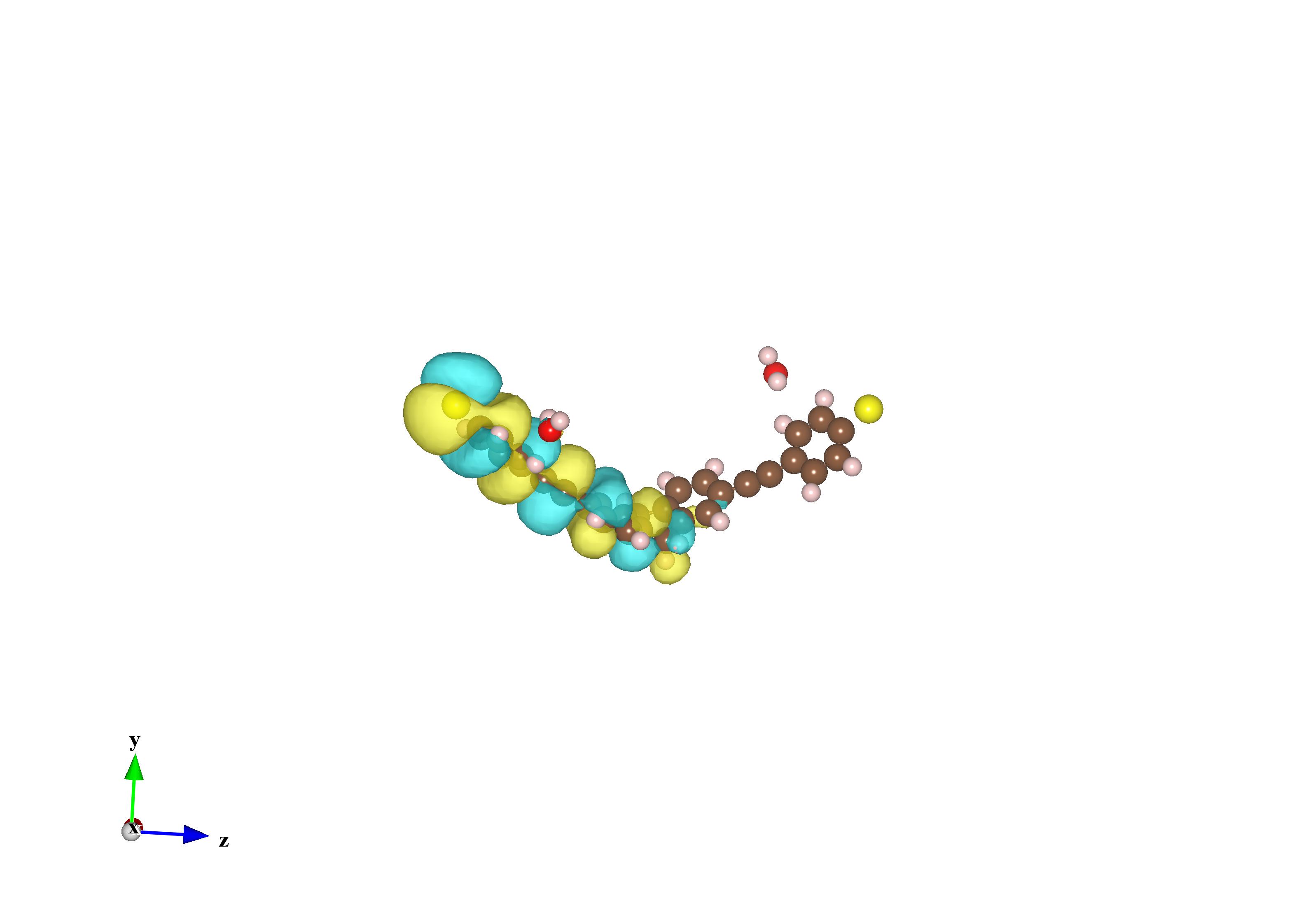 |
|  | -0.05 eV | -0.14 eV | **-0.22 eV** | -0.13 eV | -0.03 eV |
| HOMO-1 | 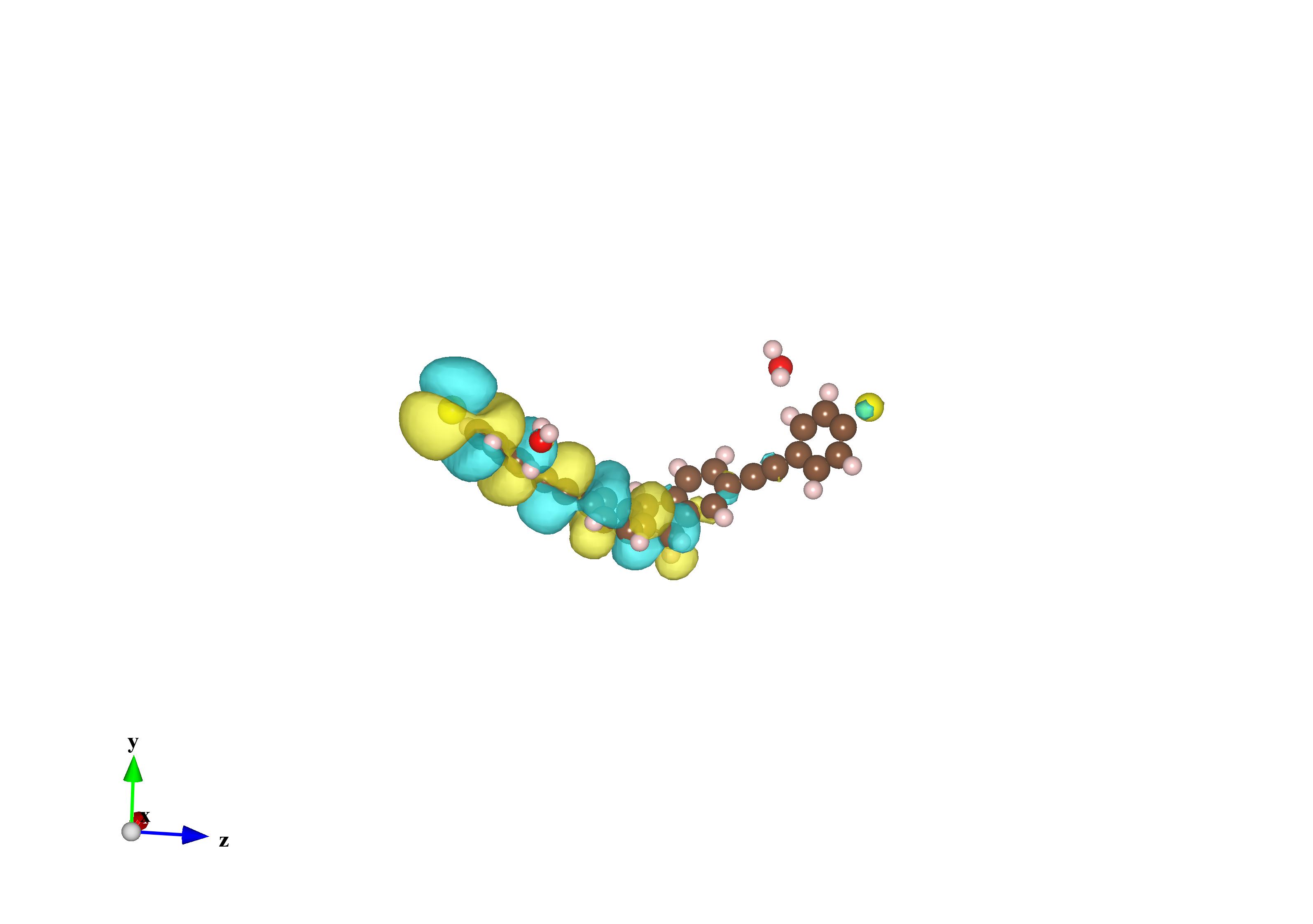 | 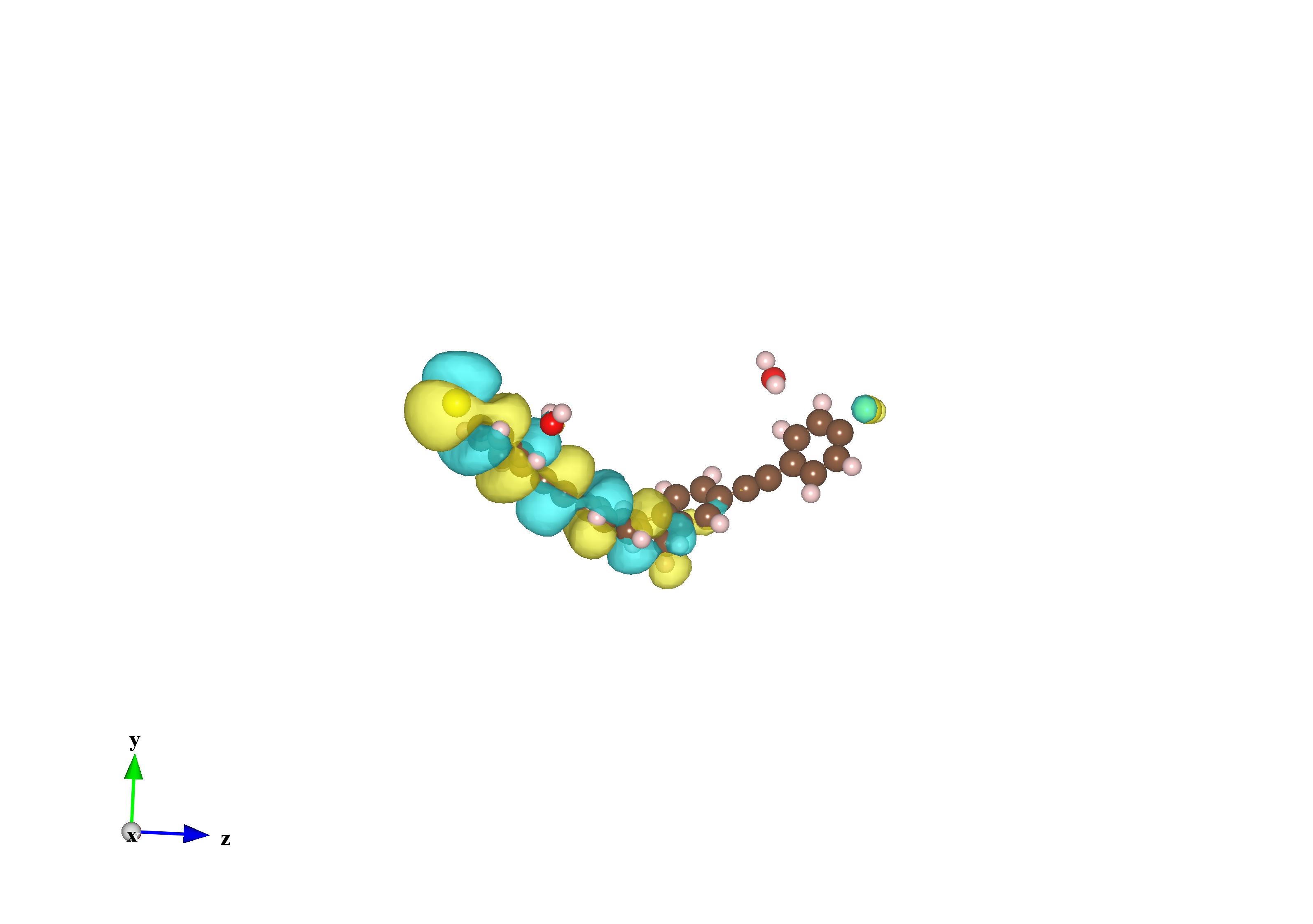 | **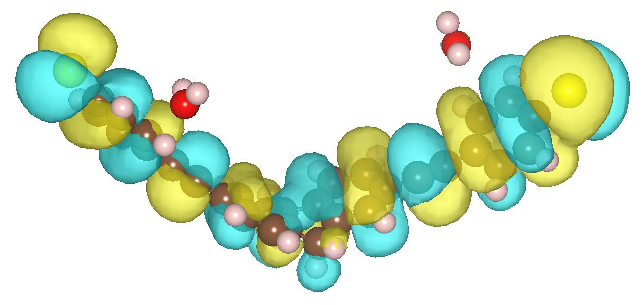** | 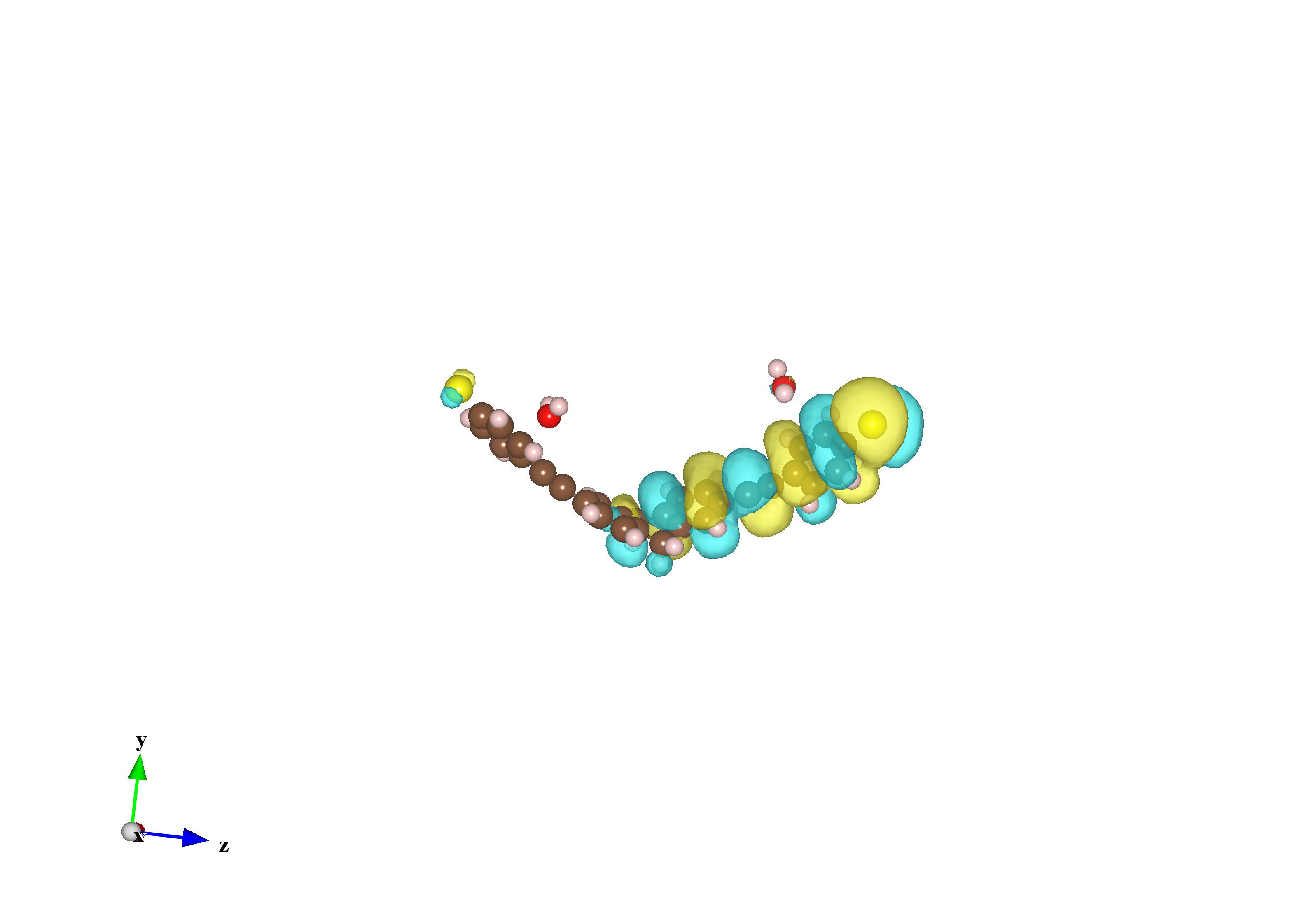 | 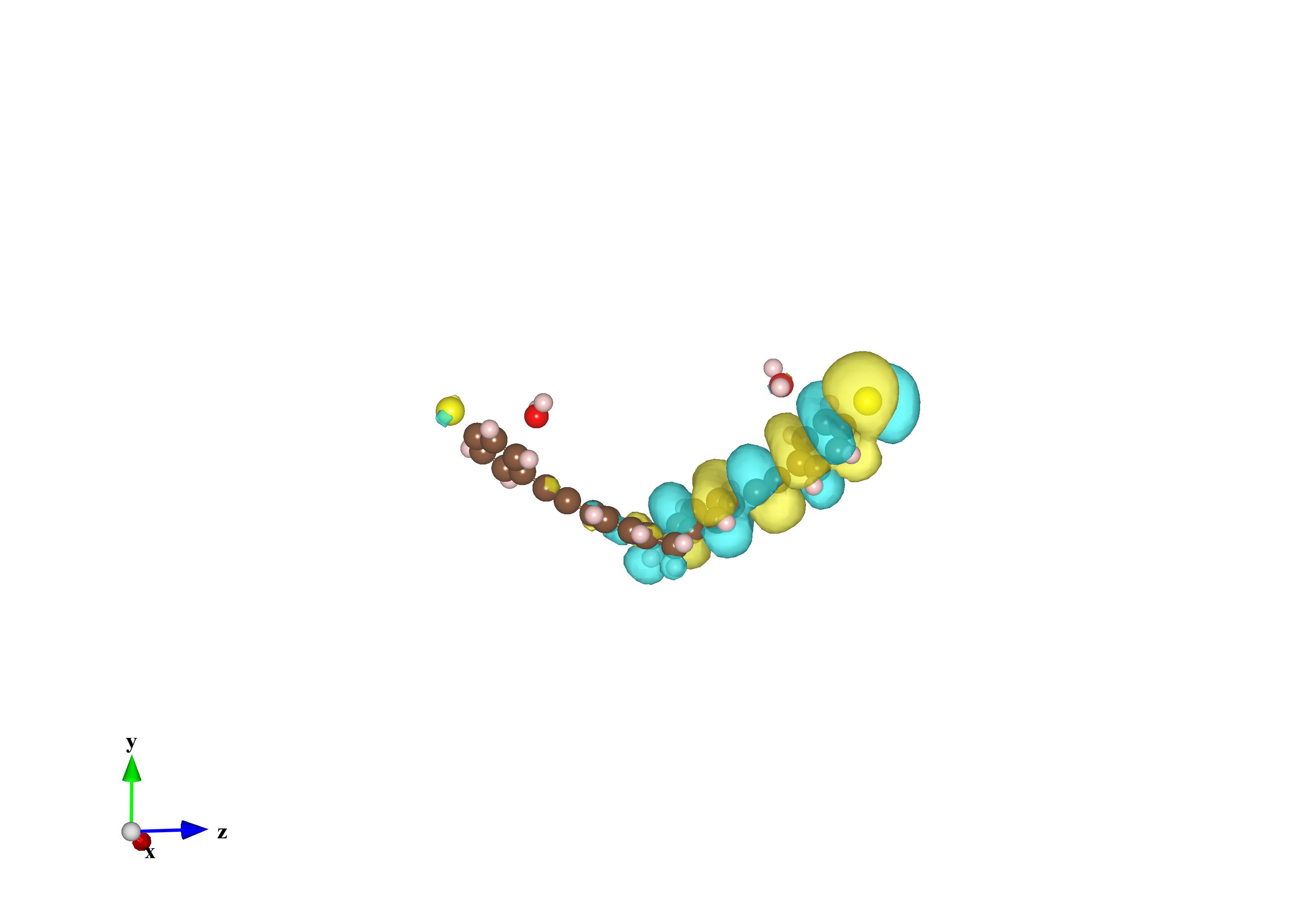 |
|  | -0.44 eV | -0.33 eV | **-0.24 eV** | -0.34 eV | -0.44 eV |
| Type II-2 | -0.50 V | -0.35V | **0.0 V** | 0.30 V | 0.50 V |
| HOMO | 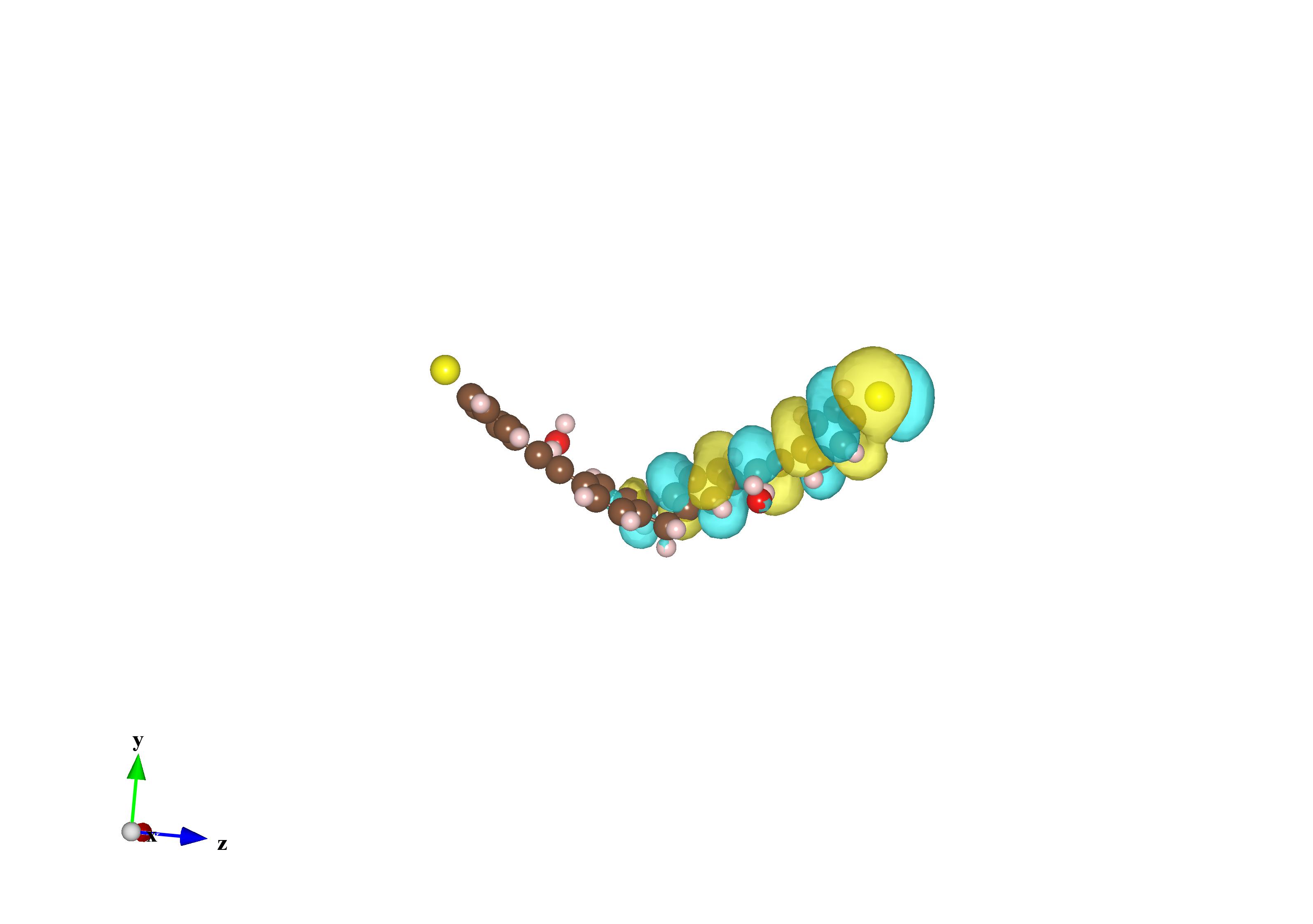 | 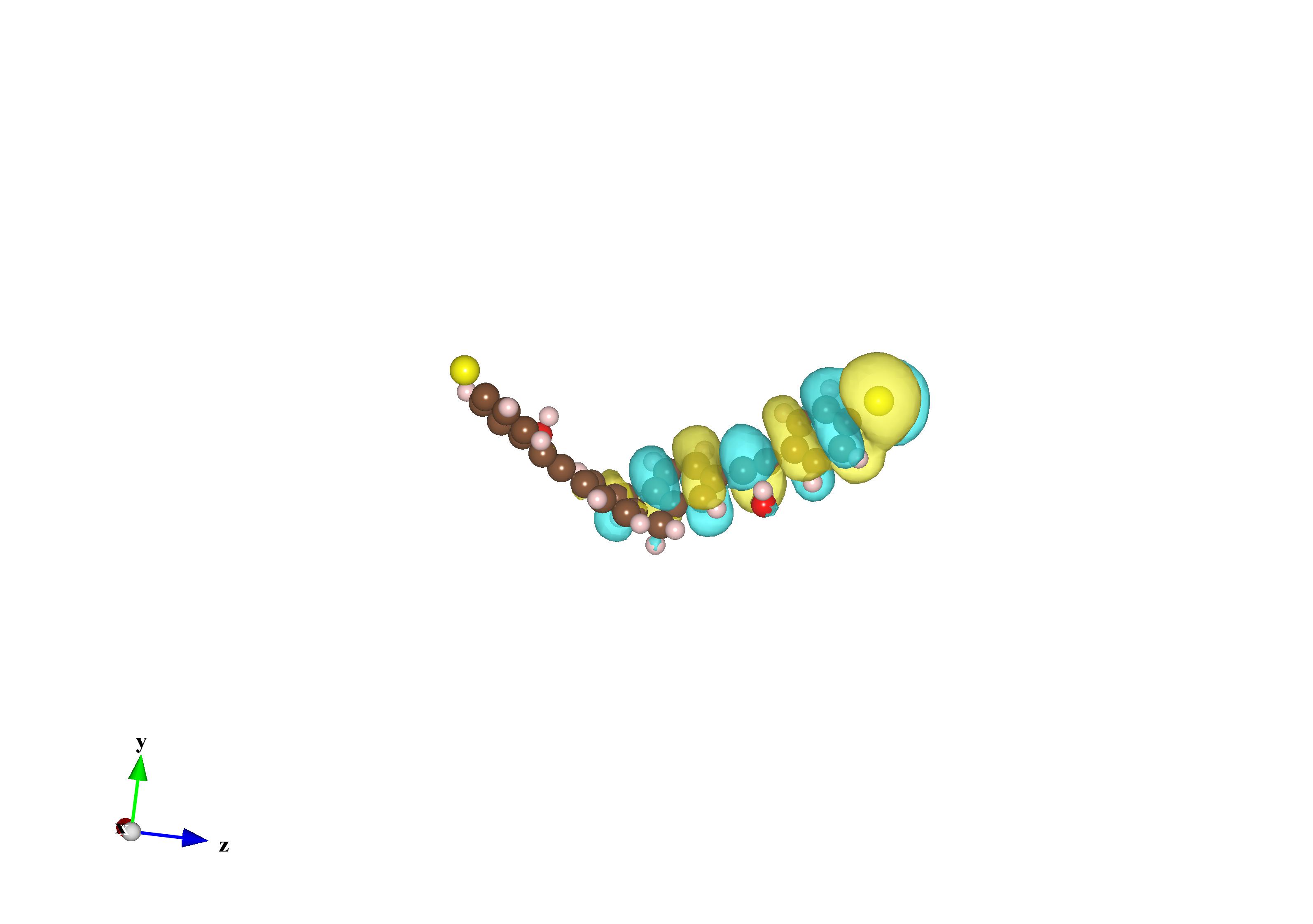 | **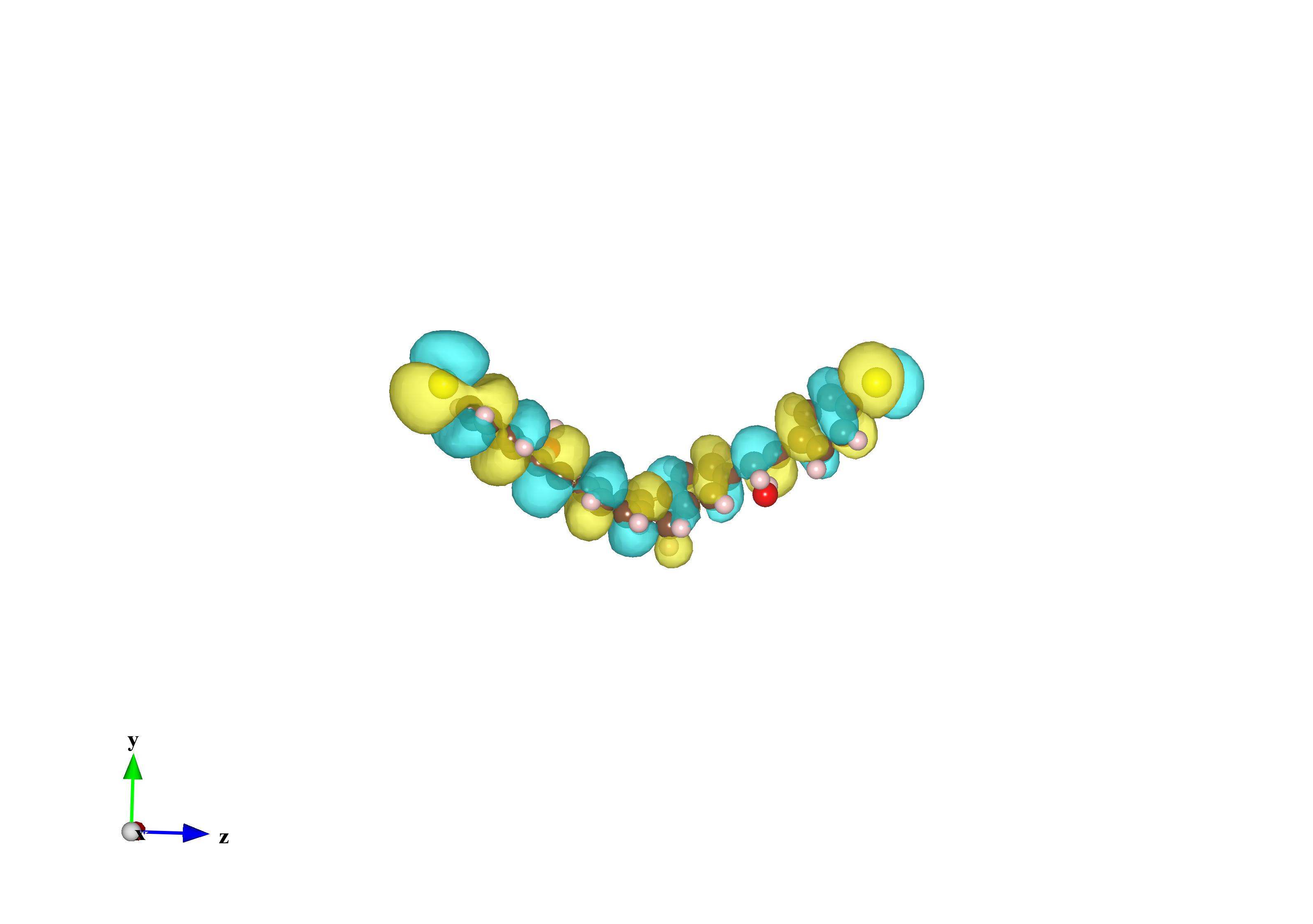** | 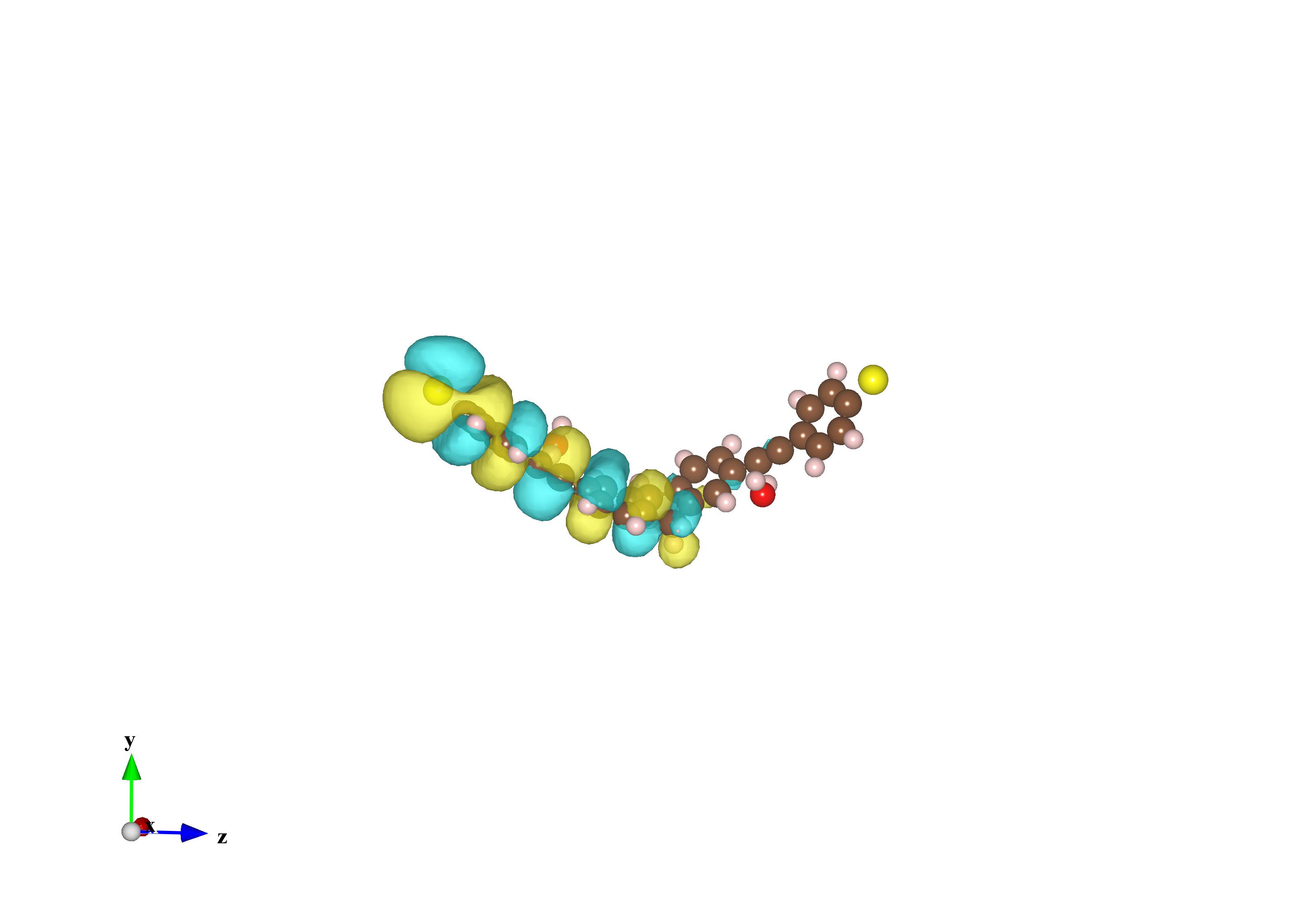 | 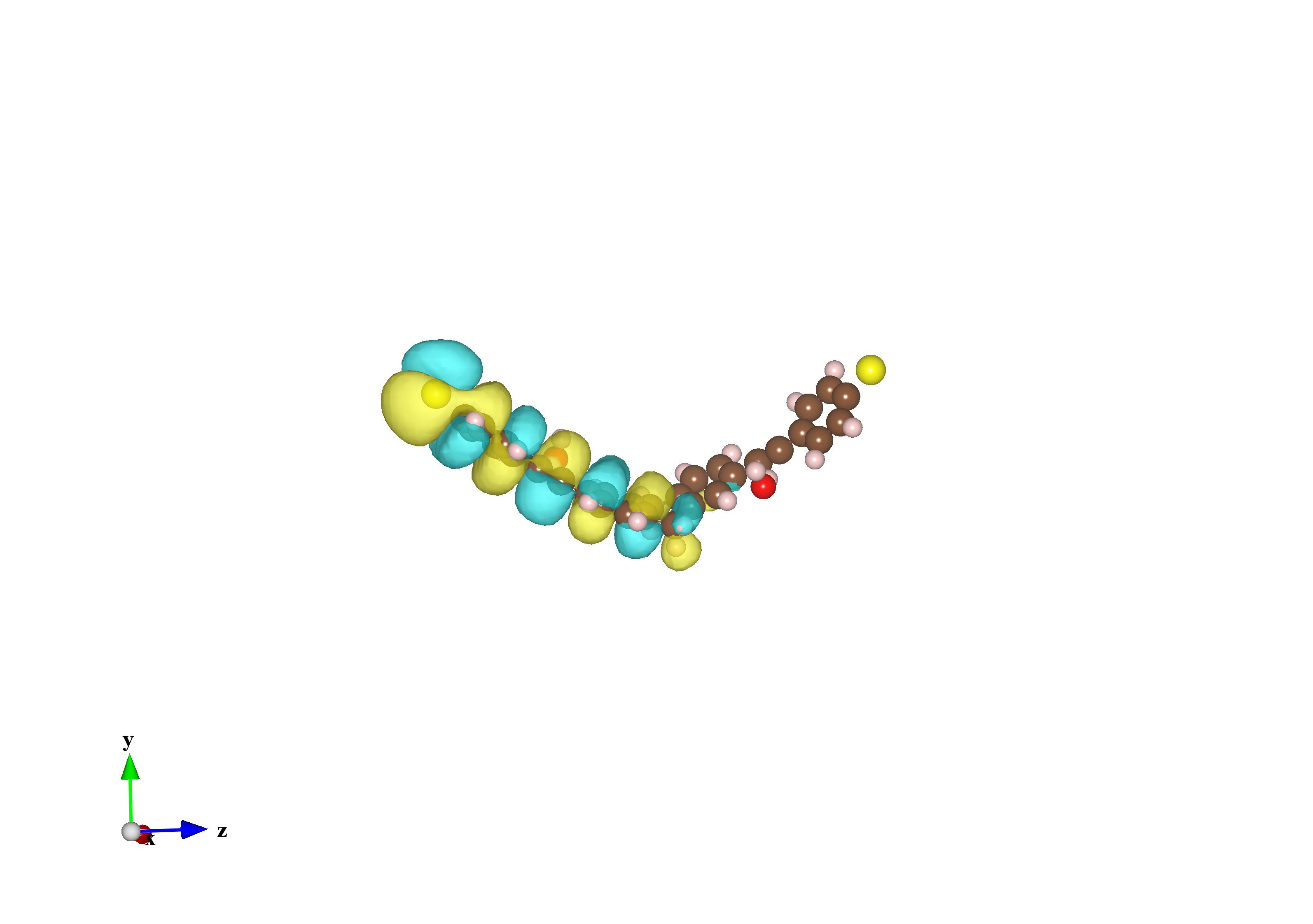 |
|  | -0.09 eV | -0.15 eV | **-0.26 eV** | -0.15 eV | -0.08 eV |
| HOMO-1 | 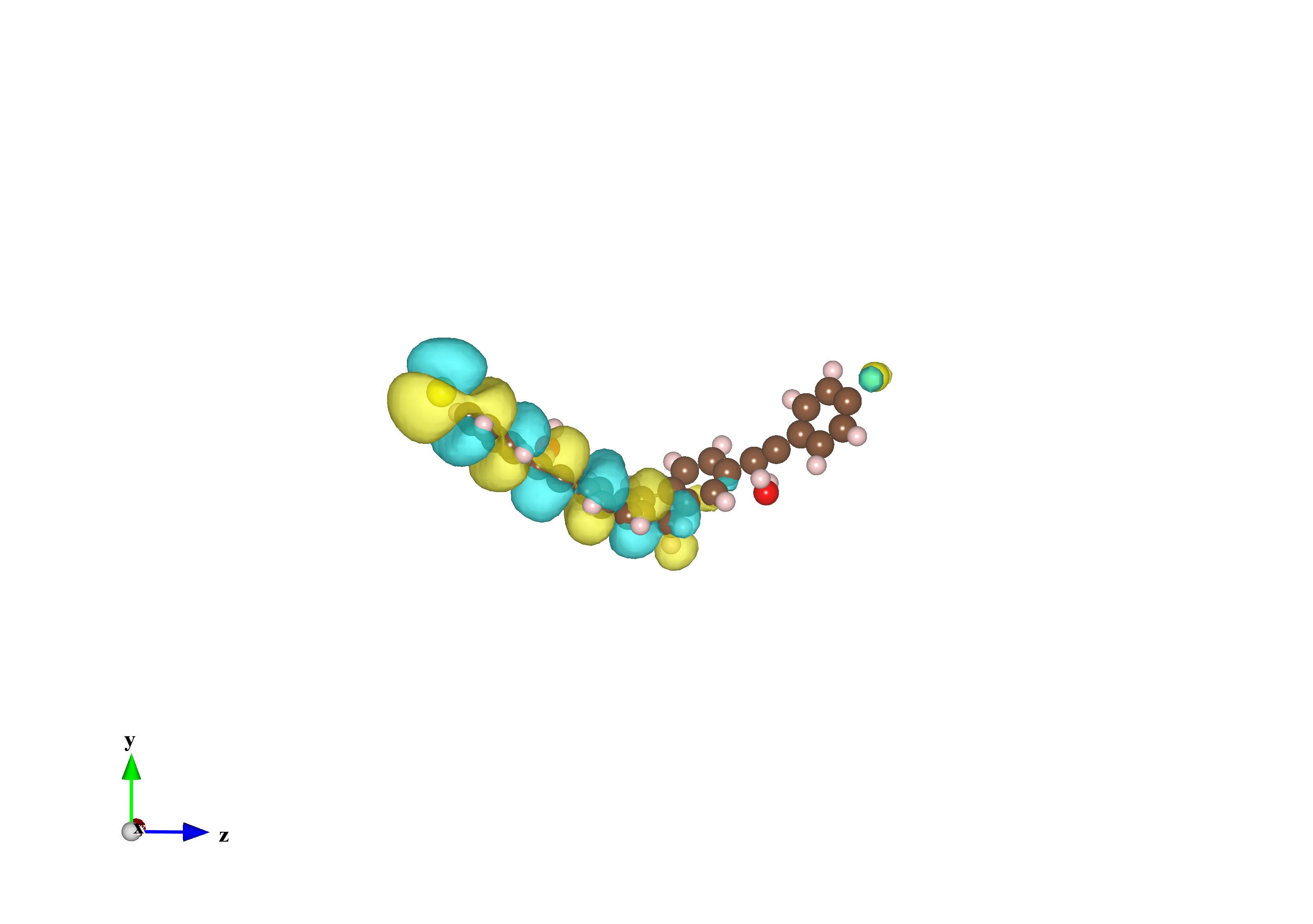  -0.47 eV | 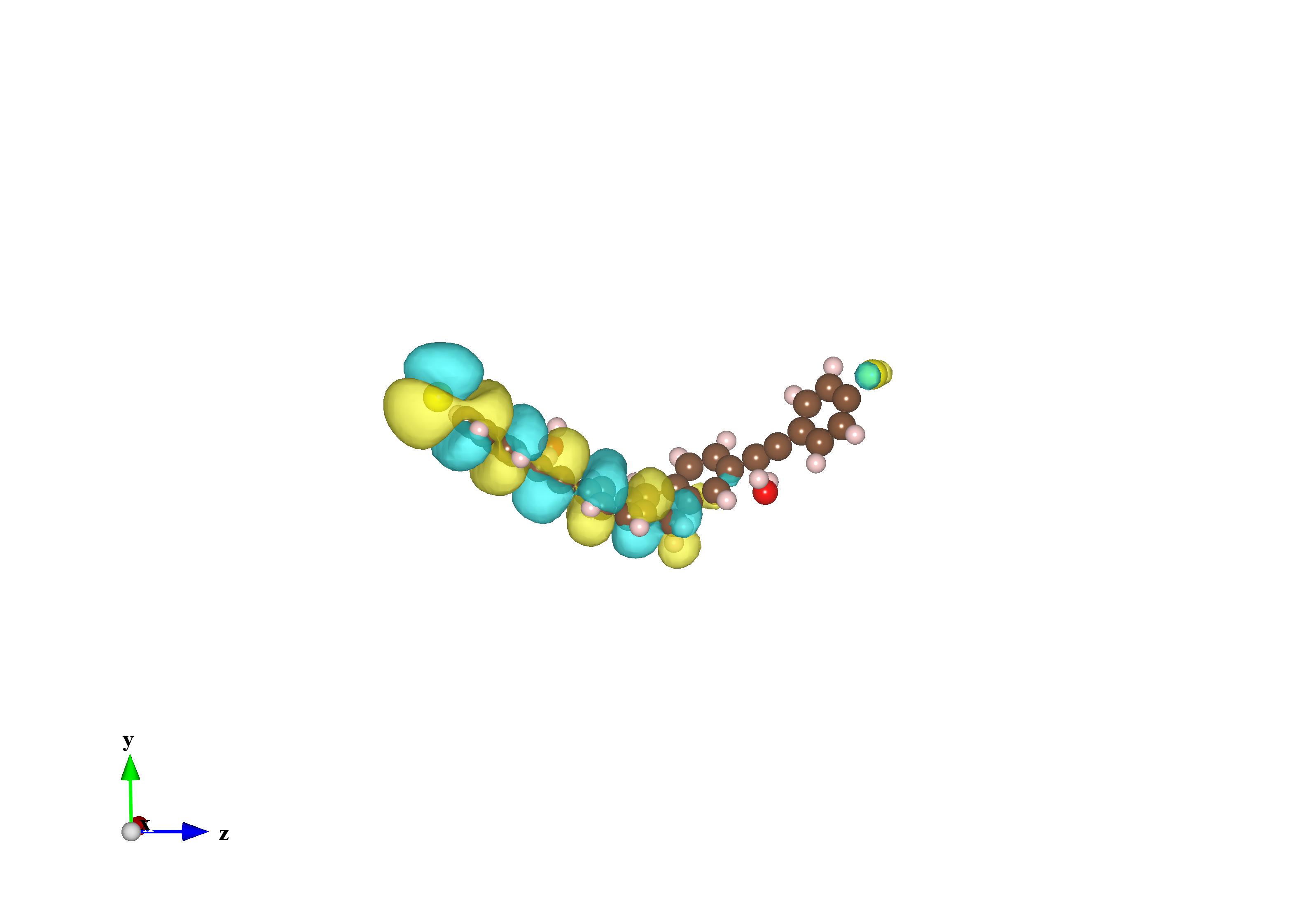  -0.40 eV | **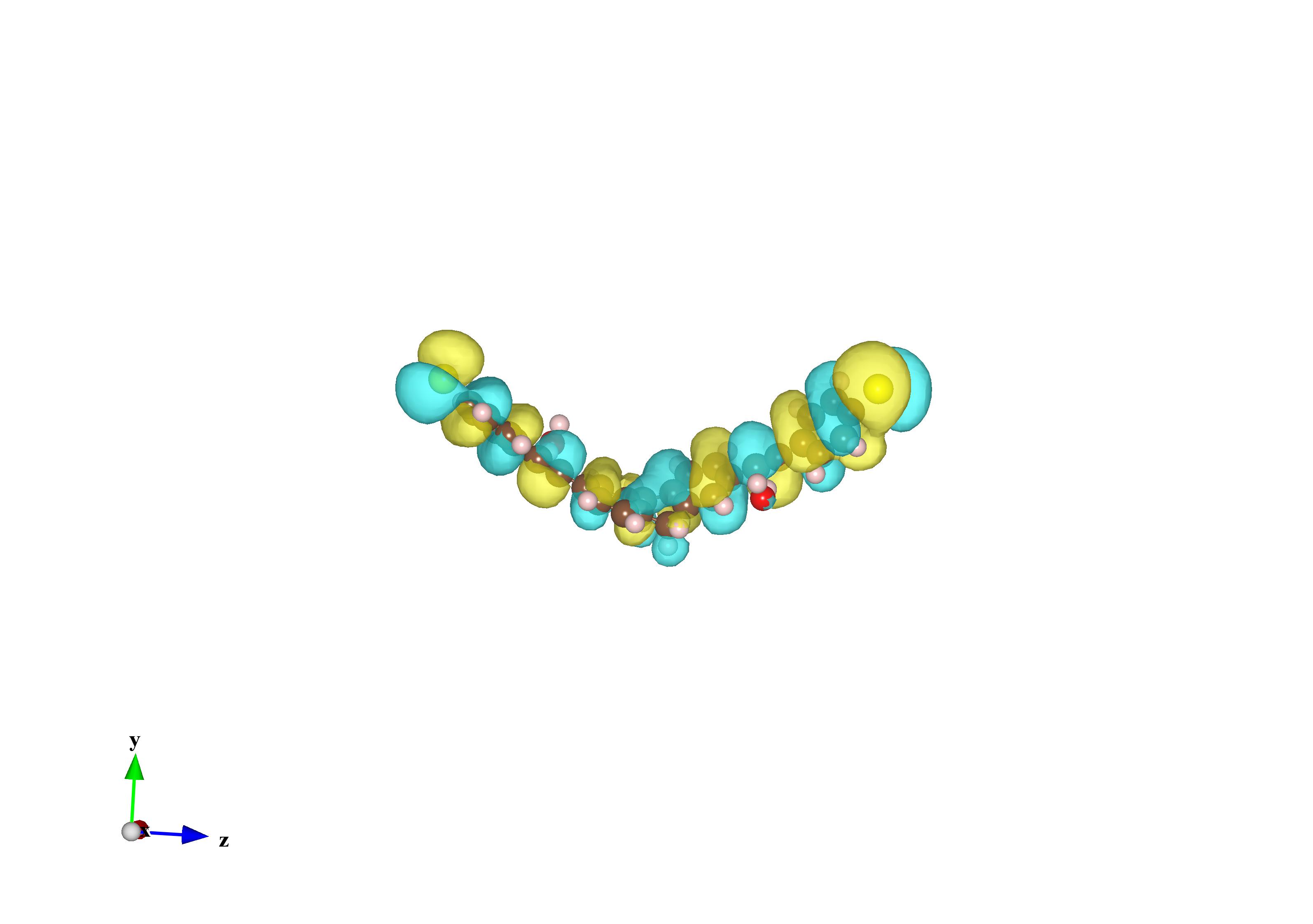**  **-0.28 eV** | 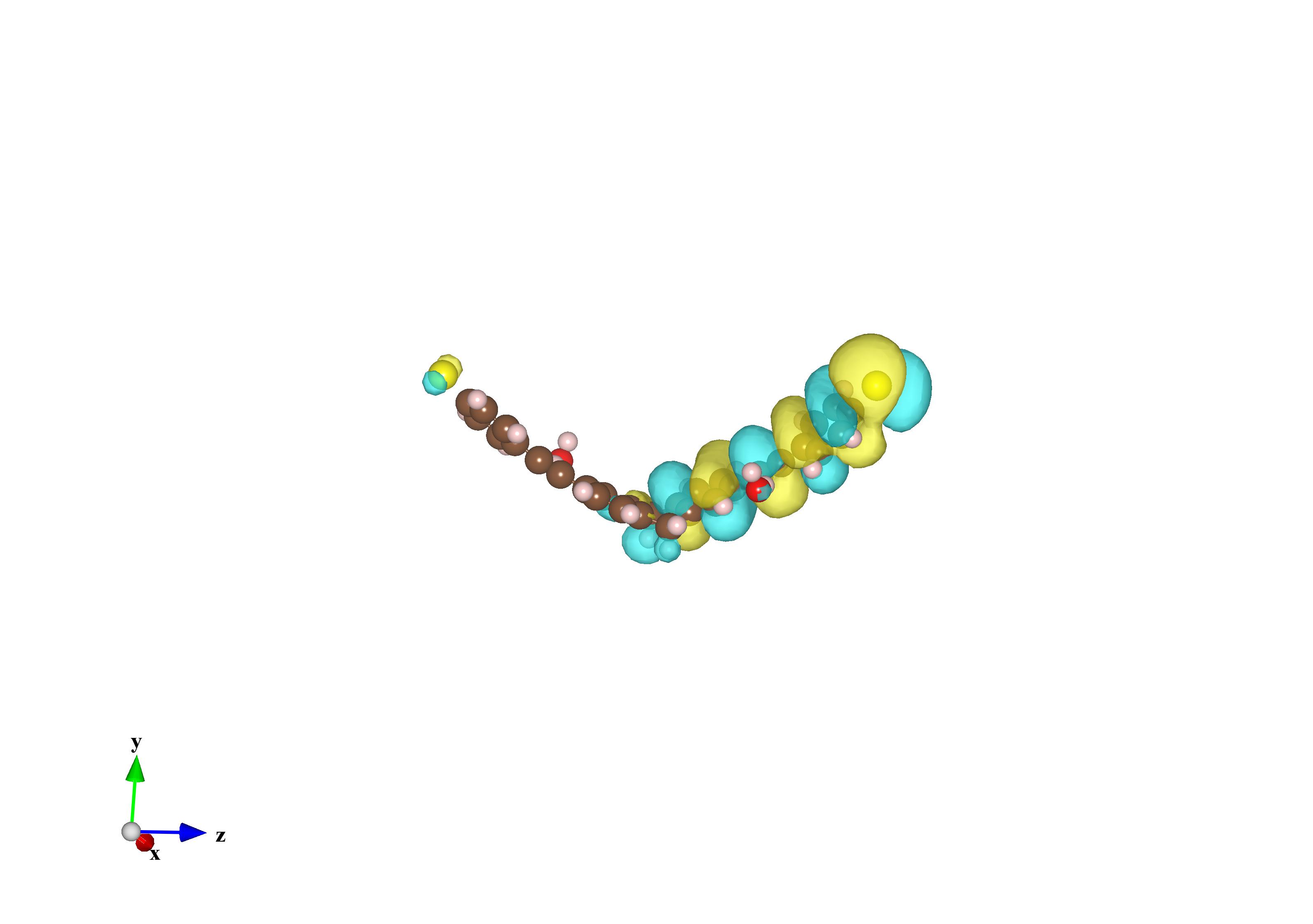  -0.39 eV | 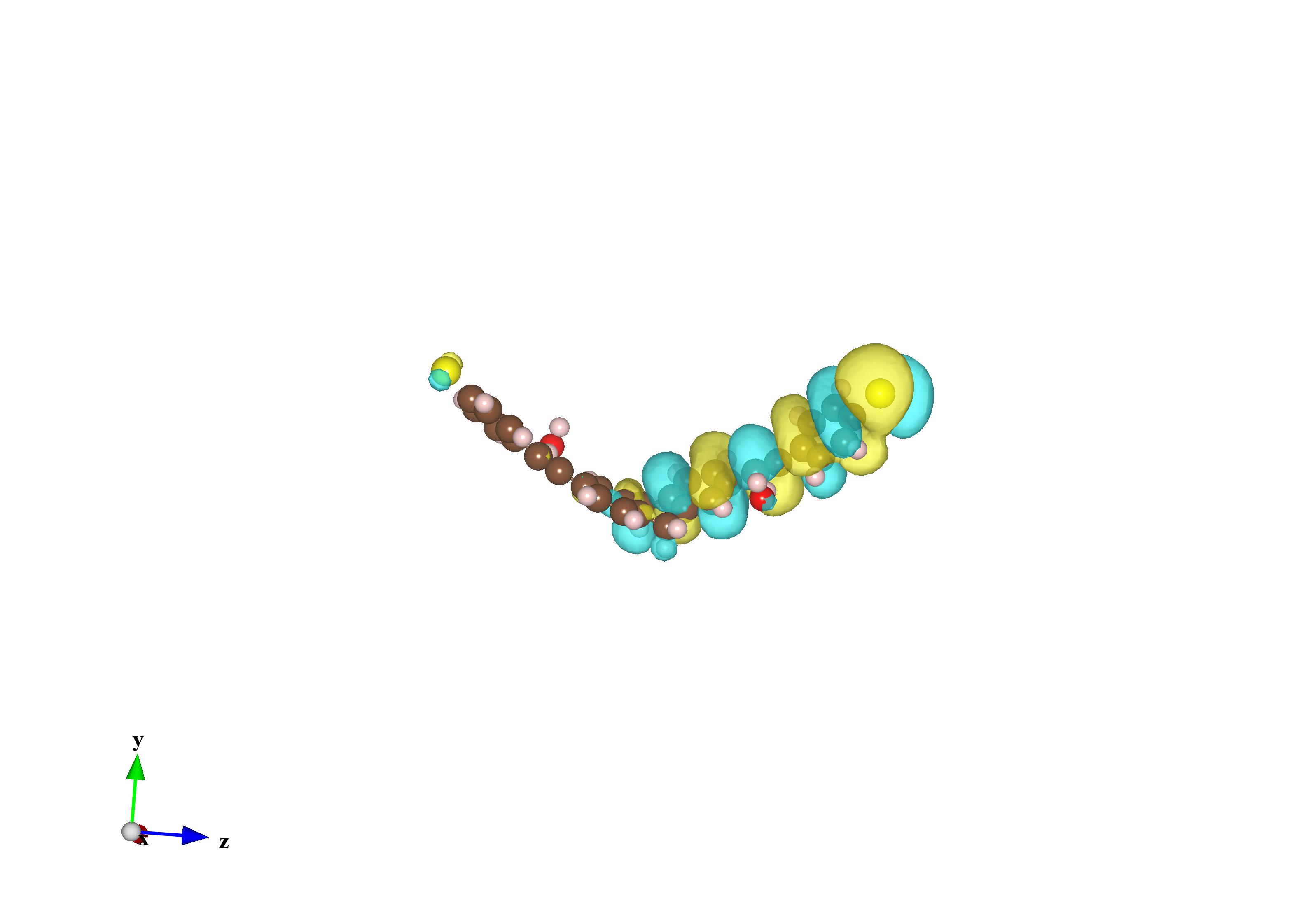  -0.47 eV |
| Type II-3 | -0.20V | **0.0 V** | 0.15 V | 0.30 V | 0.50 V |
| HOMO | 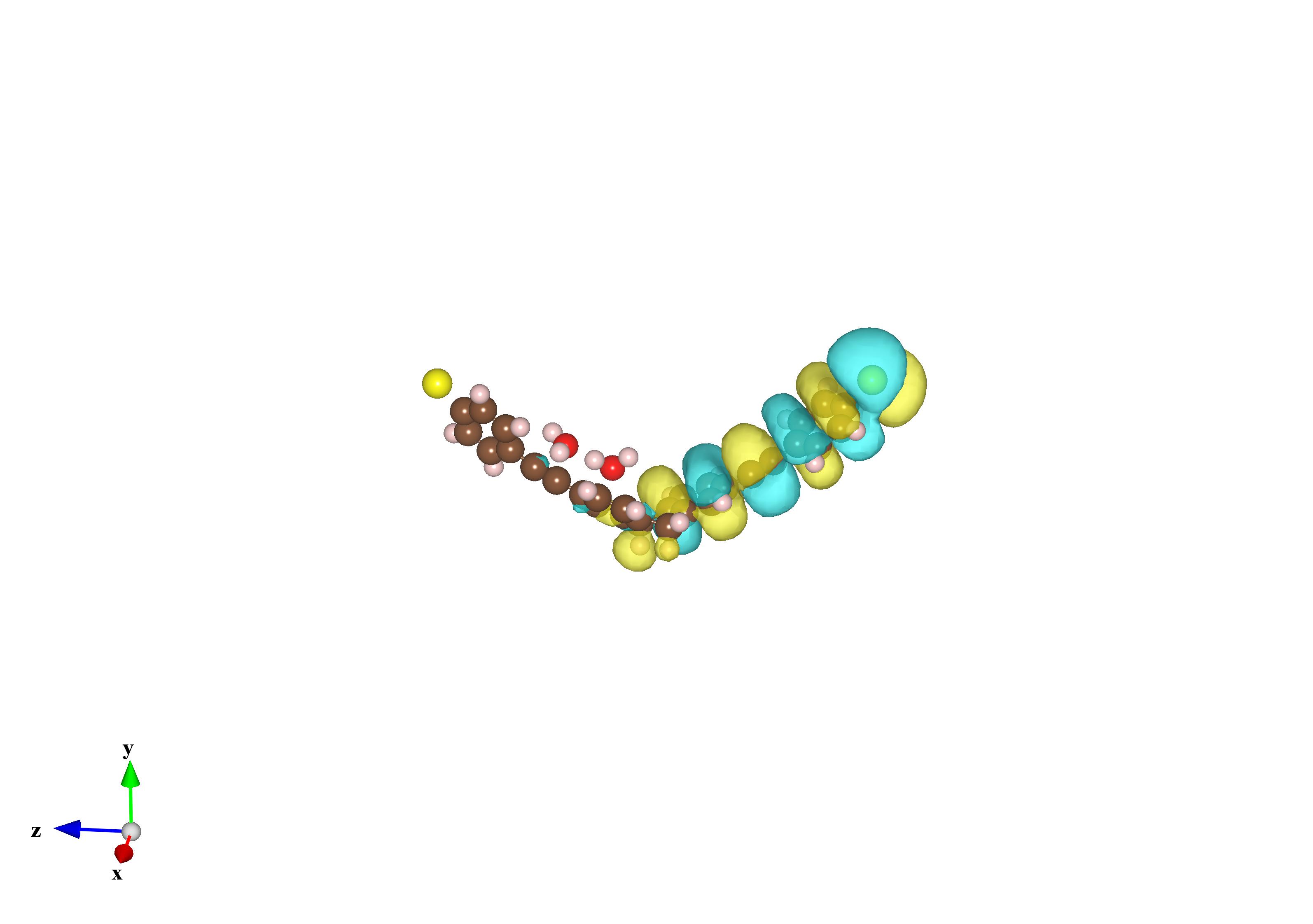 | **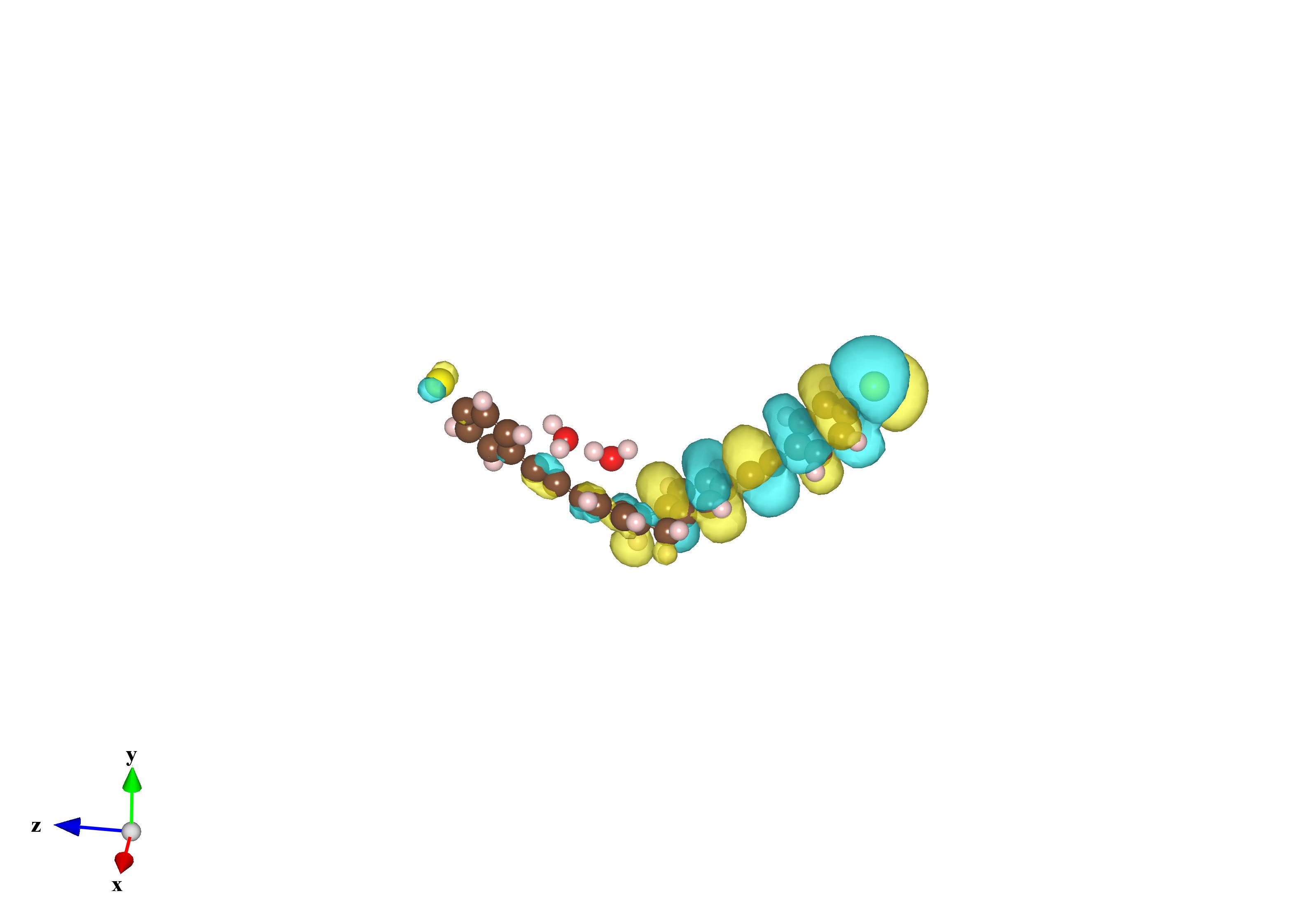** | 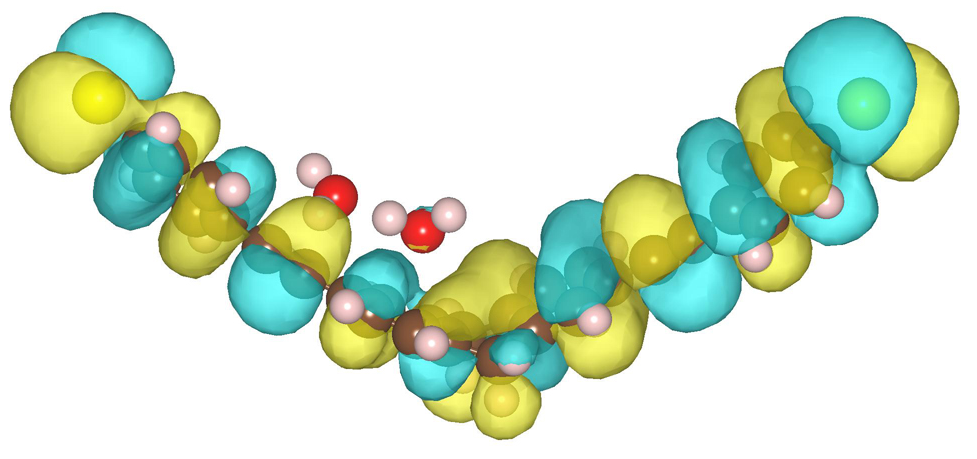 | 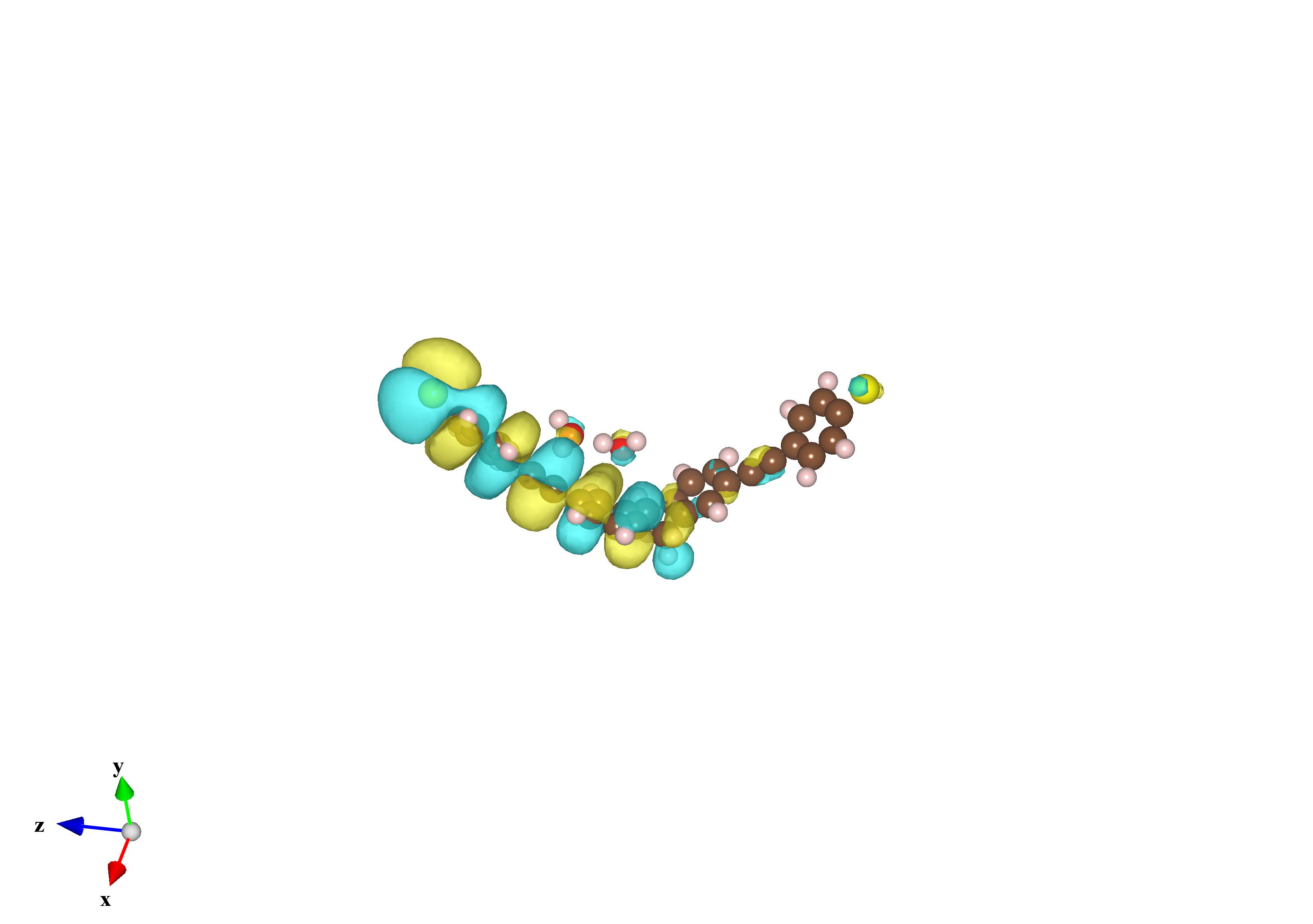 | 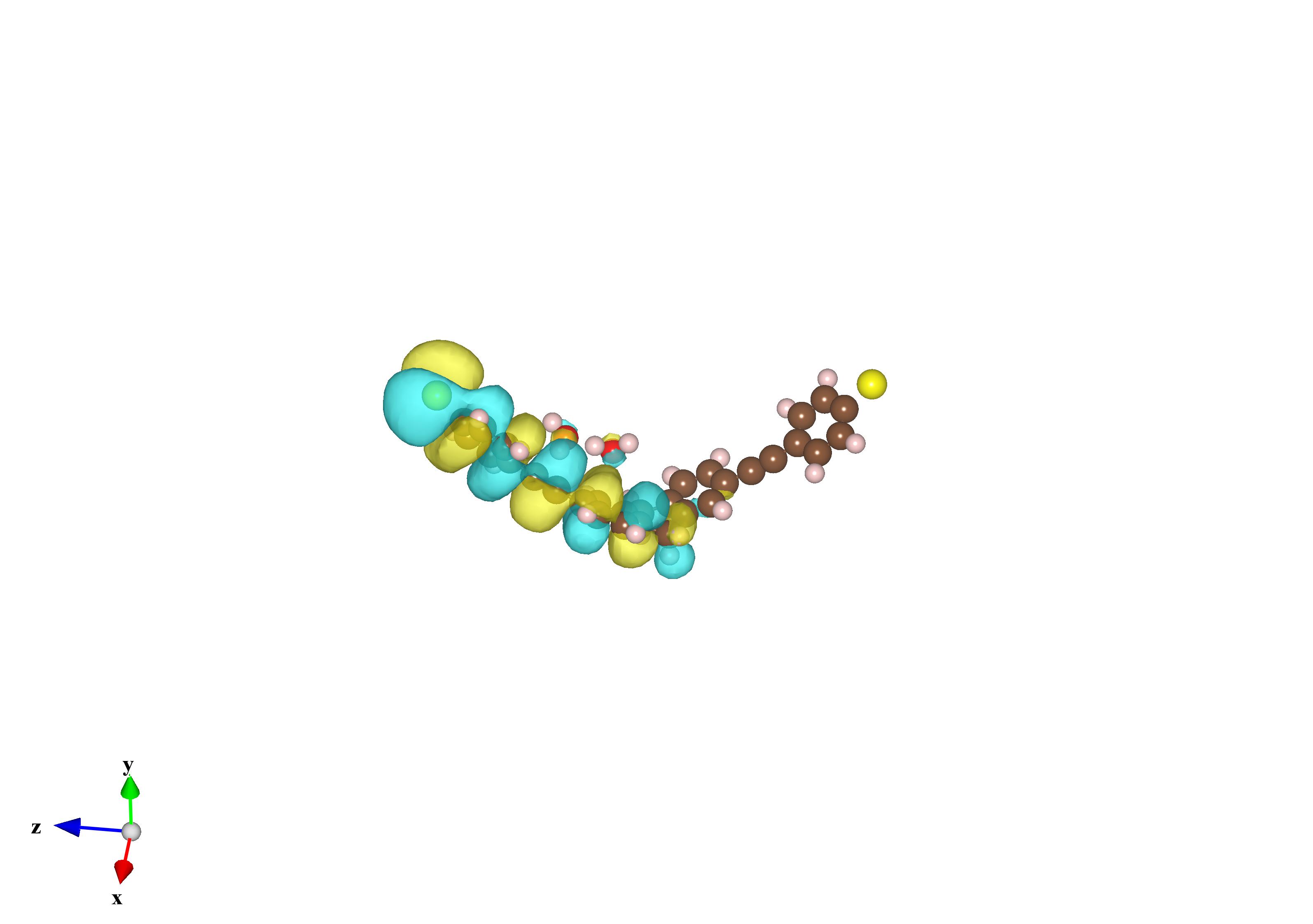 |
|  | -0.09 eV | **-0.17 eV** | -0.23eV | -0.18 eV | -0.11 eV |
| HOMO-1 | 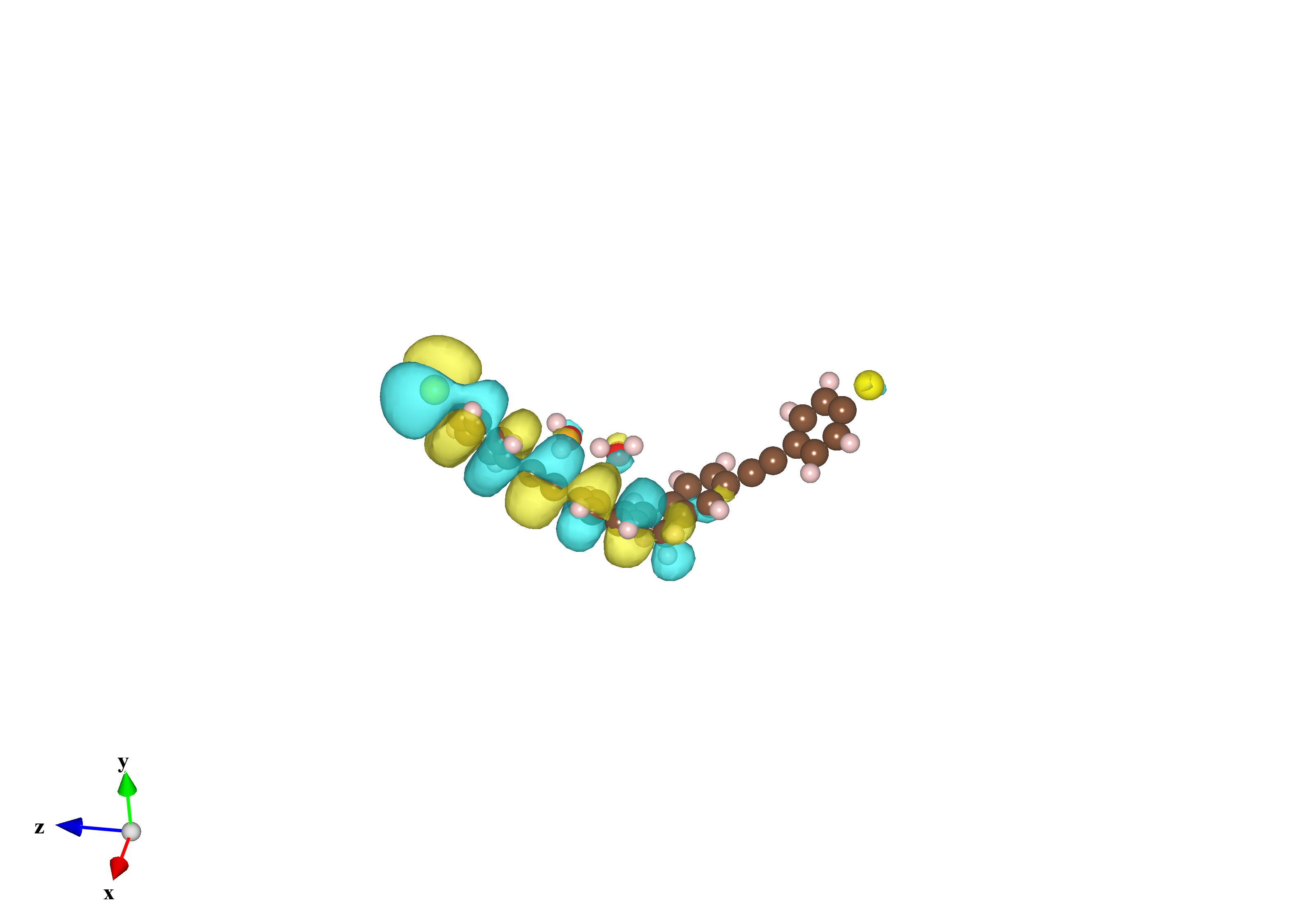 | **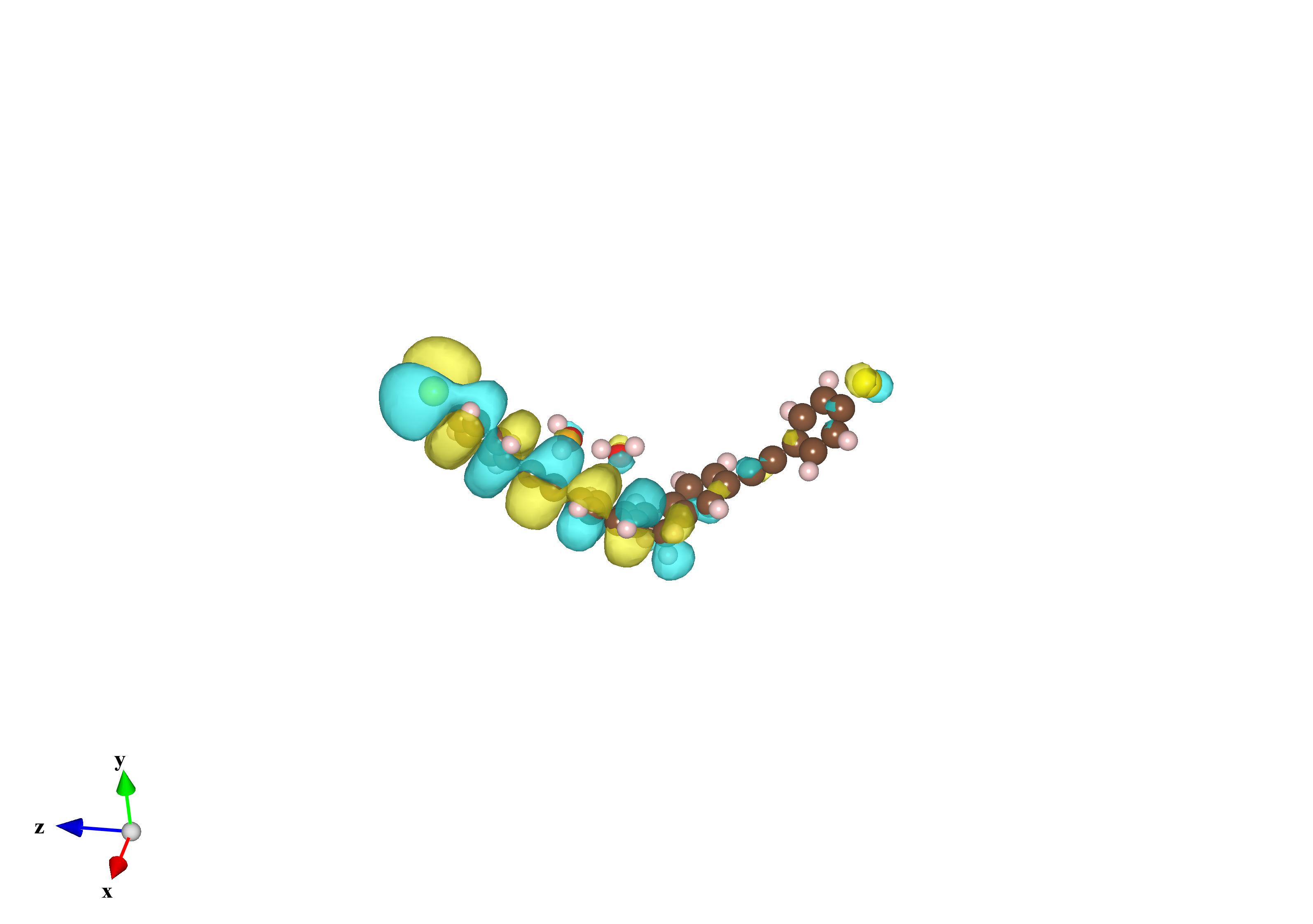** | 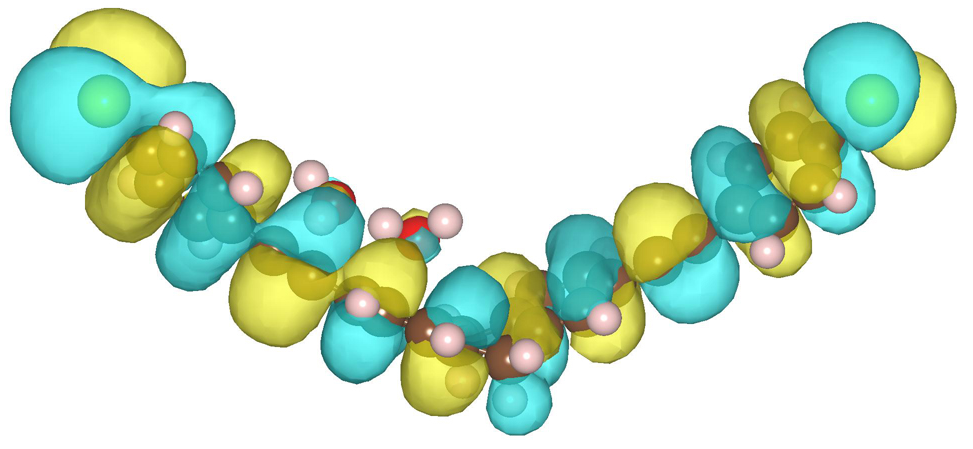 | 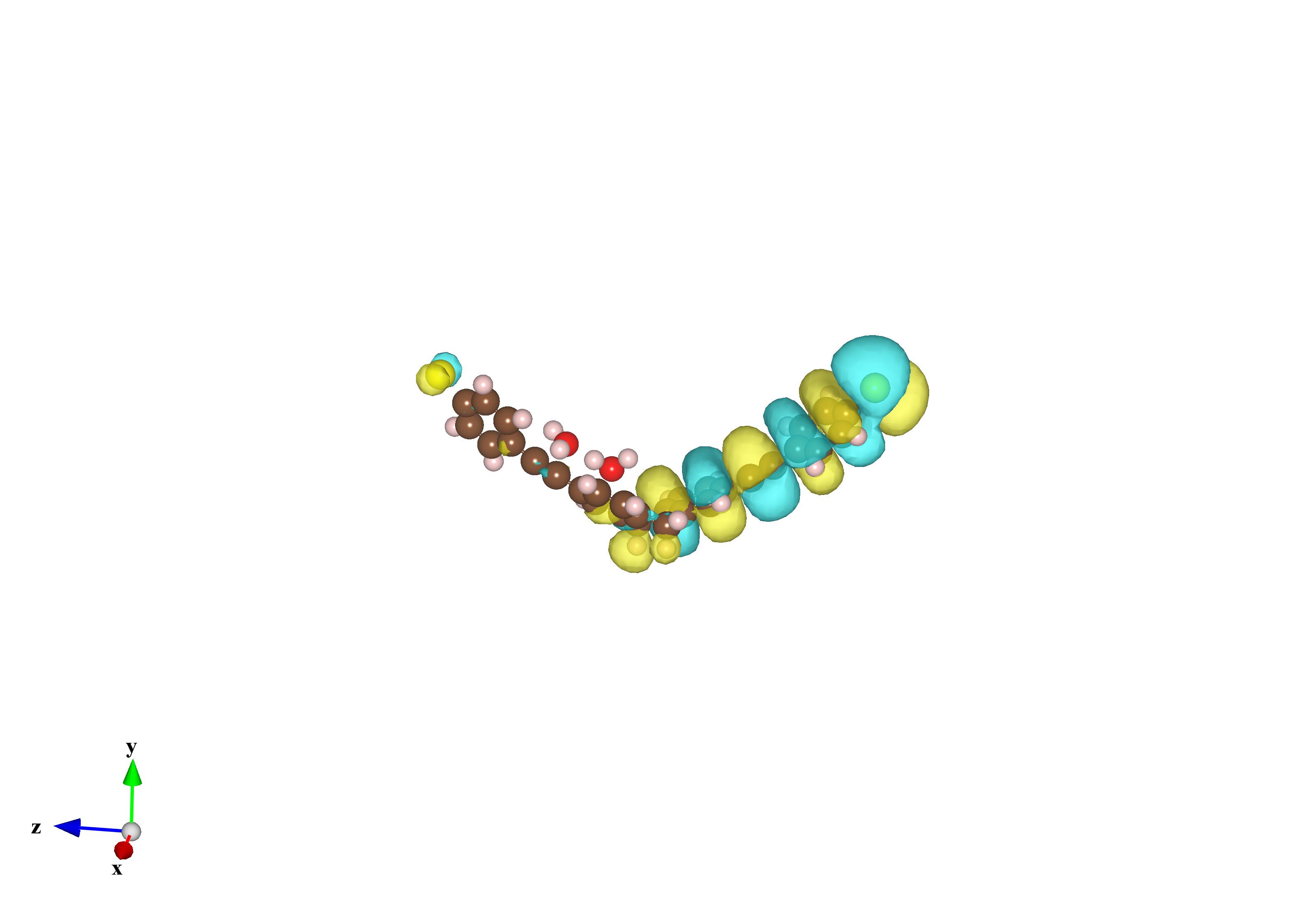 | 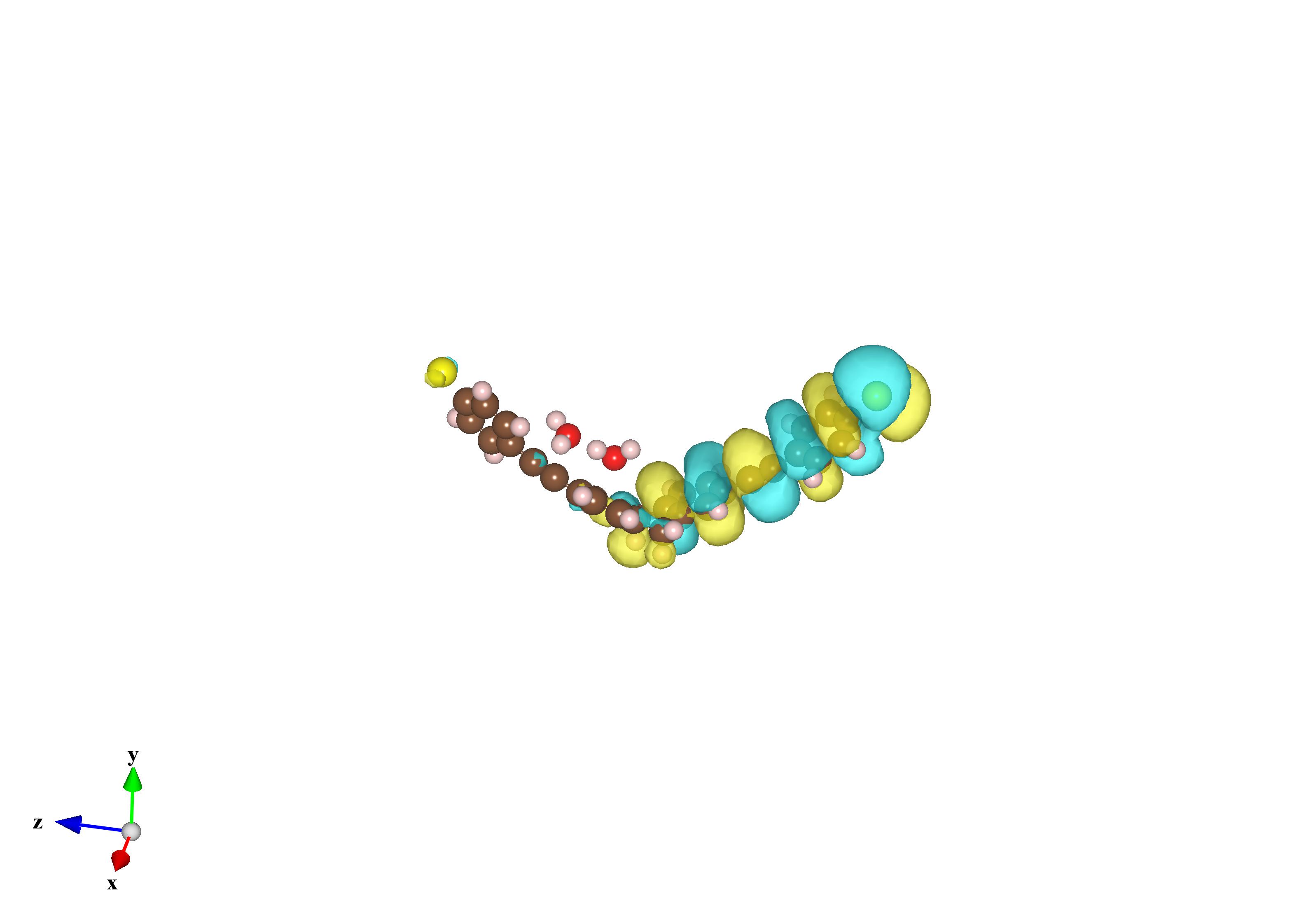 |
|  | -0.35 eV | **-0.28 eV** | -0.24eV | -0.30 eV | -0.40 eV |
| Type I-4 | -0.50 V | -0.25V | **0.0 V** | 0.25 V | 0.50 V |
| HOMO | 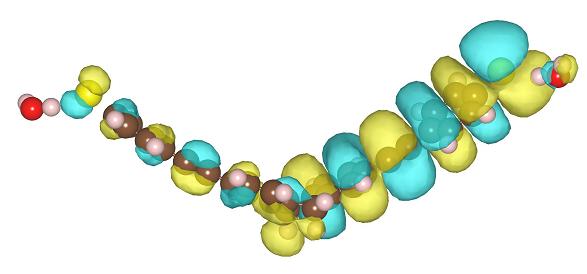 | 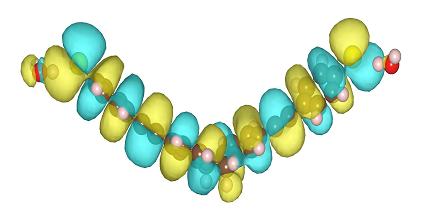 | **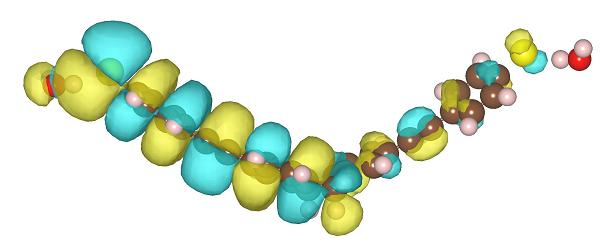** | 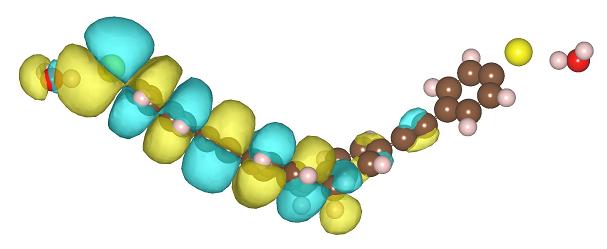 | 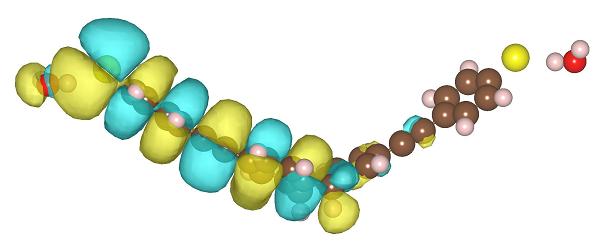 |
|  | -0.537 eV | -0.587 eV | **-0.527eV** | -0.459 eV | -0.392 eV |
| HOMO-1 | 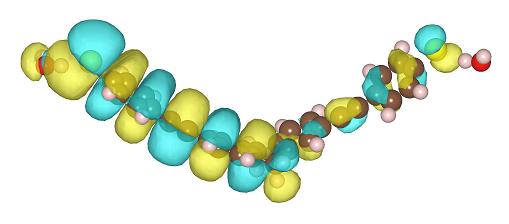 | 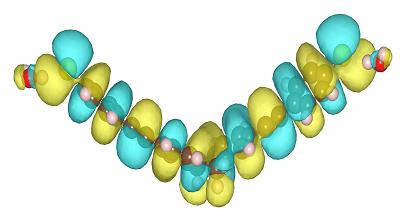 | **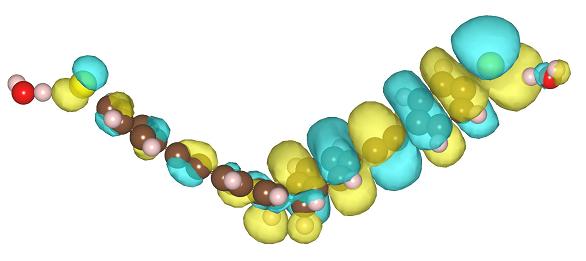** | 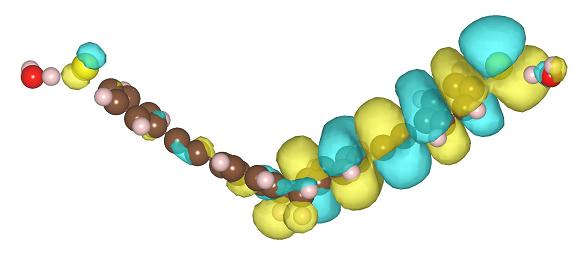 | 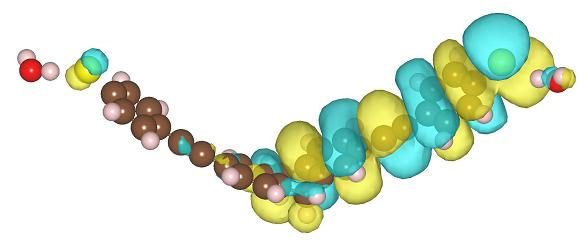 |
|  | -0.67 eV | -0.616 eV | **-0.68 eV** | -0.751 eV | -0.824 eV |

Figure S1 Molecular orbitals for TADHA molecular junctions with H_2_O molecules being adsorbed on at 0.0 V, ±0.5V and at the peak-current voltage, where the orbital energies relative to the Fermi level are also shown under each orbital.

2. **Evolution of frontier molecular orbital of TADHA molecular junctions**

Figure S2. The energy evolutions of the LUMO, HOMO and HOMO-1 as functions of bias voltage with two or three H_2_O adsorbates, the dashed line in the figure denotes bias windows
